# A damage-structured PDE model of stem cell hierarchies: The dual role of dedifferentiation in tissue homeostasis and aging

**DOI:** 10.1371/journal.pone.0335163

**Published:** 2026-02-24

**Authors:** Louis Shuo Wang, Jiguang Yu, Zonghao Liu

**Affiliations:** 1 Department of Mathematics, University of Tennessee, Knoxville, Tennessee, United States of America; 2 College of Engineering, Boston University, Boston, Massachusetts, United States of America; 3 Innovation Center for Cancer Research, Clinical Oncology School, Fujian Medical University, Fuzhou, China; Tarbiat Modares University, IRAN, ISLAMIC REPUBLIC OF

## Abstract

Stem cells maintain tissue integrity through a balance of self-renewal, differentiation, and loss of function due to aging or stress. Recent studies demonstrate that the stem cell hierarchy is not fixed. Transit-amplifying or terminally differentiated cells can dedifferentiate back into stem-like states. Such plasticity supports regeneration but, when combined with damage accumulation, may also accelerate aging and increase cancer risk.

Motivated by these findings, we develop a damage-structured PDE model of a two-compartment lineage consisting of stem and terminally differentiated cells. The model incorporates dedifferentiation, together with a nonlocal *δ*-function kernel partitioning scheme that conserves total damage and encodes biologically motivated asymmetries. Methodologically, we emphasize reproducibility and robustness on three fronts. First, the *δ*-kernel partitioning prevents the unbounded drift that arises in local models while preserving conservation. Second, a conservative finite-volume discretization with upwind fluxes and verified first-order accuracy ensures stability and exact mass balance, as confirmed by manufactured-solution tests. Third, distributional metrics and systematic parameter sweeps provide reproducible ways to quantify lineage-level damage dynamics under varying dedifferentiation and repair conditions. These analyses show that threshold-dependent and repair-modulated dedifferentiation both act as protective mechanisms: the former functions as a ‘detoxification loop’ that recycles high-damage cells, and the latter reduces the damage burden imported during dedifferentiation. Together, they mitigate aging-inducing effects. Parameter sweeps further delineate when dedifferentiation stabilizes tissue maintenance versus when it drives aging-like dynamics. Overall, our reproducible framework integrates biological insights on stem-cell plasticity and damage segregation with rigorous mathematical modeling, providing a foundation for experimental validation and therapeutic strategies targeting stem-cell aging and cancer initiation.

## Introduction

### Biological background: Plasticity, homeostasis, and aging risk

Stem cells maintain tissue integrity through a hierarchy of self-renewal and differentiation, giving rise to transient-amplifying (TA) and terminally differentiated (TD) cells that execute tissue functions [1–3]. While this hierarchy was traditionally viewed as unidirectional, recent studies demonstrate that it is highly plastic: differentiated or progenitor cells can dedifferentiate back into stem-like states [4]. This plasticity plays a dual role in tissue physiology.

On the one hand, dedifferentiation is beneficial for regeneration. It allows the tissue to replenish the stem population following injury or loss. For instance, airway epithelial cells revert to stem states after ablation [5], and melanocyte stem cells utilize dedifferentiation to support long-term maintenance [6]. Similarly, myoblasts can return to satellite cell-like states, providing a reservoir for muscle repair [7].

On the other hand, this backward flow can be detrimental when combined with damage accumulation. Aging naturally disrupts the balance of self-renewal and attrition, leading to the accumulation of mutations and cellular dysfunction in long-lived cells [8]. When damaged TD cells dedifferentiate, they may reintroduce this functional damage into the stem population, effectively “importing” risk. For example, dedifferentiation-derived neural stem cells have been reported to carry functional damage that may promote dysfunction [9]. Mechanisms such as asymmetric organelle inheritance during divisions [10] and evolutionary trade-offs between segregation and repair [11] provide mechanisms for damage segregation and further shape outcomes. While in cancer, shifts in division symmetry drive malignancy [12].

### Modeling rationale and contribution

Mathematical modeling has long been used to study stem cell dynamics [13]. Approaches range from multi-compartmental ODE frameworks that capture tumor growth and treatment resistance through stem-non-stem interactions [14,15] to hybrid methods estimating mutation timelines [16]. Other studies focus on feedback regulation, mutant clone evolution, and how transient dynamics shape heterogeneity and resistance [17–19]. However, theoretical predictions diverge on the role of dedifferentiation in fixation probabilities of neutral mutations [20,21]. Beyond cancer, models address stem cell aging, homeostasis, and damage segregation. They include ODE formulations of regulatory stability [22–25], PDE transport and age-structured models [26–29], and delay equations capturing oscillatory cell-cycle regulation [30–33]. Recent PDE approaches further link damage accumulation, segregation, and feedback control to long-term population fitness [34]. They provide a foundation for understanding how intrinsic damage dynamics interact with tissue-level homeostasis. However, a significant gap remains. Most existing frameworks treat lineages as unidirectional or do not explicitly track how inherited damage interacts with dedifferentiation.

To bridge this gap, we developed a damage-structured PDE model of a two-compartment lineage consisting of stem and TD cells. Unlike standard age-structured models, our structuring variable tracks accumulated dysfunction rather than chronological time. Key features of our approach include: (1) a nonlocal inheritance mechanism where damage is partitioned during division; (2) flexible dedifferentiation rules that allow us to test constant, threshold-dependent, and repair-modulated scenarios; (3) feedback regulation on proliferation and dedifferentiation; and (4) a conservative finite-volume scheme with verified convergence and mass conservation. This framework allows us to rigorously investigate specific hypotheses: Under what conditions does dedifferentiation rescue tissue maintenance? When does it conversely accelerate damage accumulation, driving an aging-like phenotype? And critically, how do protective mechanisms like damage thresholds and partial repair alter these outcomes?

## Methods

### Biological framing and modeling assumptions

To bridge the gap between biological plasticity and mathematical formalism, we first define the biological interpretation of our model compartments and the nature of the damage variable before presenting the governing equations.

The model simplifies the lineage into two primary compartments. The first, denoted by *P*, represents the stem-like pool. This compartment encompasses not only long-lived “true” stem cells but also cycling progenitor cells capable of self-renewal. The second compartment, *W*, represents the TD pool responsible for tissue function. While intermediate TA cells exist in vivo, we simplify the hierarchy to focus on the regulatory feedback between the stem and TD populations. This reduction allows us to explicitly track the flow of damage during dedifferentiation without the added complexity of intermediate stages.

The structuring variable *x* represents the accumulation of intracellular dysfunction rather than chronological age or cell-cycle time. Biologically, this abstracts various deleterious factors, such as protein aggregates, organelle defects, and DNA lesions. A cell with a higher *x* is not necessarily older in time but is functionally more compromised and prone to apoptosis.

We investigate three distinct biological hypotheses regarding how plasticity operates:

(i) **Constant dedifferentiation** represents indiscriminate plasticity, where TD cells revert to the stem state at a fixed rate regardless of their damage burden.
(ii) **Threshold-dependent dedifferentiation (TDD)** represents a damage-triggered reprogramming mechanism. Here, we hypothesize that dedifferentiation is a stress response activated only when cellular damage exceeds a critical threshold *x* = *x*_*c*_, potentially acting as a mechanism to recycle or repair failing cells.
(iii) **Partial repair** tests the hypothesis that the reprogramming process itself induces rejuvenation. In this scenario, the act of dedifferentiating clears a portion of the accumulated damage, returning the cell to the stem population in a healthier state than it left.

### Mathematical model

We first develop the mathematical framework. Following the framework, we present PDEs that describe coupled stem and TD populations under damage-structured dynamics and nonlocal partitioning. We then impose boundary conditions and feedback regulations to close the system, reflecting biological control mechanisms. Finally, we clarify the simplifying assumptions underlying the model and explain how they may be relaxed.

#### Variables, compartments, and damage structuring.

We begin with the PDE system that underpins the analysis. The model captures the dual role of dedifferentiation. This role can either mitigate or exacerbate damage. The framework combines advection–reaction dynamics with nonlocal partitioning. It also incorporates feedback-regulated transitions between stem and TD cells.

The system consists of two populations: the stem cell *P*(*t*,*x*) and the TD cell *W*(*t*,*x*), where t≥0 is time and x≥0 is a structuring variable for cumulative damage. Stem cells divide with three possible outcomes: self-renewal with probability *p*_1_, differentiation with probability *p*_2_, or asymmetric division with probability *p*_3_. The coupled PDEs governing stem and TD populations are

$$\left\{ \begin{array}{cc} \partial_{t}P+v_{P}\partial_{x}P & =\int_{0}^{\infty }p_{1}\lambda_{P}K_{1,P}(x,x^{\prime })P(t,x^{\prime })\;dx^{\prime }\\ & +\int_{0}^{\infty }p_{3}\lambda_{P}K_{3,P}(x,x^{\prime })P(t,x^{\prime })\;dx^{\prime }-\lambda_{P}P+\lambda_{R}W,\\ \partial_{t}W+v_{W}\partial_{x}W & =\int_{0}^{\infty }p_{2}\lambda_{P}K_{2,W}(x,x^{\prime })P(t,x^{\prime })\;dx^{\prime }\\ & +\int_{0}^{\infty }p_{3}\lambda_{P}K_{3,W}(x,x^{\prime })P(t,x^{\prime })\;dx^{\prime }-(\delta (x)+\lambda_{R})W. \end{array}\right.$$ (1)

Here, $v_{P}$ and $v_{W}$ are constant damage accumulation rates, and the kernels $K_{\cdot ,\cdot }$ encode nonlocal inheritance of damage during replication [35,36].

#### Division modes and dedifferentiation mechanisms.

Stem replication follows nonlocal partitioning rules. These rules ensure the conservation of total damage. There are three types of division in this model:

(i) Symmetric self-renewal (P→P+P): this process assigns damage fractions $\alpha_{1}$ and $\alpha_{2}$ to the daughters. The sum of these fractions $\alpha_{1}+\alpha_{2}$ equals 1.
(ii) Symmetric differentiation (P→W+W): this division assigns damage fractions $\beta_{1}$ and $\beta_{2}$ to the new cells. The total $\beta_{1}+\beta_{2}$ also equals 1.
(iii) Asymmetric division (P→P+W): the stem daughter receives damage fraction $\gamma_{1}$. The TD daughter receives damage fraction $\gamma_{2}$. These fractions $\gamma_{1}+\gamma_{2}$ sum to 1.

Baseline choices are $\alpha_{1}=\alpha_{2}=0.5$, $\beta_{1}=\beta_{2}=0.5$, $\gamma_{1}=1/3$, $\gamma_{2}=2/3$, ensuring that each division conserves total damage. Asymmetric partitioning reflects experimental observations that the stem daughter inherits less damage than the TD daughter [37,38]. These rules are represented by *δ*-function transition kernels [34]:


$$K_{1,P}(x,x^{\prime })=\delta (x-\alpha_{1}x^{\prime })+\delta (x-\alpha_{2}x^{\prime }),\;K_{2,W}(x,x^{\prime })=\delta (x-\beta_{1}x^{\prime })+\delta (x-\beta_{2}x^{\prime }),\;K_{3,P}(x,x^{\prime })=\delta (x-\gamma_{1}x^{\prime }),\;K_{3,W}(x,x^{\prime })=\delta (x-\gamma_{2}x^{\prime }).$$

Substituting these kernels into Eq (1) yields the advection-reaction system

$$\left\{ \begin{array}{cc} \partial_{t}P+v_{P}\partial_{x}P & =\frac{p_{1}\lambda_{P}}{\alpha_{1}}P(t,x/\alpha_{1})+\frac{p_{1}\lambda_{P}}{\alpha_{2}}P(t,x/\alpha_{2})\\ & +\frac{p_{3}\lambda_{P}}{\gamma_{1}}P(t,x/\gamma_{1})+\lambda_{R}W-\lambda_{P}P,\\ \partial_{t}W+v_{W}\partial_{x}W & =\frac{p_{2}\lambda_{P}}{\beta_{1}}P(t,x/\beta_{1})+\frac{p_{2}\lambda_{P}}{\beta_{2}}P(t,x/\beta_{2})\\ & +\frac{p_{3}\lambda_{P}}{\gamma_{2}}P(t,x/\gamma_{2})-(\delta (x)+\lambda_{R})W, \end{array}\right.$$ (2)

Here, $v_{P},v_{W}>0$ denote damage accumulation rates, $\lambda_{P}>0$ is the stem replication rate, $\lambda_{R}\geq 0$ is the dedifferentiation rate, and δ(x)≥0 is the damage-dependent TD cell death rate. The prefactors 1/α for $\alpha \in \{\alpha_{i},\beta_{i},\gamma_{i}\}$ follow from the substitution property of the *δ*-function:

$$\delta (x-\alpha x^{\prime })=\frac{1}{\left|\alpha \right|}\delta (x^{\prime }-\frac{x}{\alpha }).$$ (3)

The formulation above assumes that dedifferentiation preserves damage exactly. More generally, dedifferentiation can be modeled by a transition kernel $r(x|x^{\prime })=\delta (x\;-\;\rho x^{\prime })$ with retention fraction ρ∈[0,1]. The kernel $r(x|x^{\prime })$ maps a TD cell with damage *x*^′^ to a stem cell with reduced damage $x\leq x^{\prime }$. The framework covers three cases: full retention with ρ=1 in Eq (2), partial repair with 0<ρ<1, and complete repair with ρ=0. Substituting $r(x|x^{\prime })$ into Eq (1) yields the more general system:

$$\left\{ \begin{array}{cc} \partial_{t}P+v_{P}\partial_{x}P & =\frac{p_{1}\lambda_{P}}{\alpha_{1}}P(t,x/\alpha_{1})+\frac{p_{1}\lambda_{P}}{\alpha_{2}}P(t,x/\alpha_{2})\\ & +\frac{p_{3}\lambda_{P}}{\gamma_{1}}P(t,x/\gamma_{1})+\frac{\lambda_{R}}{\rho }W(t,x/\rho )-\lambda_{P}P,\\ \partial_{t}W+v_{W}\partial_{x}W & =\frac{p_{2}\lambda_{P}}{\beta_{1}}P(t,x/\beta_{1})+\frac{p_{2}\lambda_{P}}{\beta_{2}}P(t,x/\beta_{2})\\ & +\frac{p_{3}\lambda_{P}}{\gamma_{2}}P(t,x/\gamma_{2})-\frac{\lambda_{R}}{\rho }W(t,x/\rho )-\delta (x)W(t,x). \end{array}\right.$$ (4)

For analytical tractability, we focused in this paper on the conservative full-retention case ρ=1. In the Results section, we used numerical experiments to examine how partial repair with 0<ρ<1, including the representative case ρ=0.5, alters damage accumulation and outcomes.

For comparison, consider a simpler advection-reaction system without partitioning:

$$\left\{ \begin{array}{cc} \partial_{t}P+\partial_{x}(v_{P}P) & =(2f-1)\lambda_{P}P+\lambda_{R}W,\\ \partial_{t}W+\partial_{x}(v_{W}W) & =(2-2f)\lambda_{P}P-(\delta (x)+\lambda_{R})W, \end{array}\right.$$ (5)

which yields the same dynamics of total counts $\left(\bar{P},\bar{W}\right)$ as Eq (2):

$$\left\{ \begin{array}{cc} \frac{d\bar{P}}{dt} & =(2f-1)\lambda_{P}\bar{P}+\lambda_{R}\bar{W},\\ \frac{d\bar{W}}{dt} & =(2-2f)\lambda_{P}\bar{P}-\lambda_{R}\bar{W}-\int_{0}^{\infty }\delta (x)W(t,x)\;dx. \end{array}\right.$$ (6)

Here, $\bar{P}:=\int_{0}^{\infty }P(t,x)\;dx,\;\bar{W}:=\int_{0}^{\infty }W(t,x)\;dx$ are total number of stem and TD cells at time *t*, respectively. $f=(1\;+\;p_{1}\;-\;p_{2})/2$ is the renewal fraction. These expressions follow by integrating the PDEs and using the boundary conditions described next.

#### Boundary conditions and feedback regulations.

Building on the PDE system above, we next introduce the boundary conditions and feedback terms that regulate growth and ensure biological plausibility. We impose a Dirichlet boundary condition

P(t,0)=0,W(t,0)=0,t≥0,P(t,x)=0,W(t,x)=0,for x→∞, (7)

which mathematically specifies that the stem density vanishes at zero damage.

Feedback mechanisms are incorporated via negative regulation of stem replication and division probabilities by the TD population, consistent with experimental evidence [39–42]. In addition, we introduce the suppression of dedifferentiation by stem cell abundance [5]. Specifically,

$$p_{1}(t)=p_{1}(\bar{W}(t)):=\frac{{\bar{p}}_{1}}{1+(k_{1}\bar{W}(t){)}^{m_{1}}},$$ (8)

$$p_{2}(t)=p_{2}(\bar{W}(t)):=\frac{{\bar{p}}_{2}}{1+(k_{2}\bar{W}(t){)}^{m_{2}}},$$ (9)

$$\lambda_{P}(t)=\lambda_{P}(\bar{W}(t)):=\frac{{\bar{\lambda }}_{P}}{1+(k_{3}\bar{W}(t){)}^{m_{3}}},$$ (10)

$$\lambda_{R}(t)=\lambda_{R}(\bar{P}(t)):=\frac{{\bar{\lambda }}_{R}}{1+(k_{4}\bar{P}(t){)}^{m_{4}}}.$$ (11)

Here, ${\bar{p}}_{1}$, ${\bar{p}}_{2}$, ${\bar{\lambda }}_{P}$, ${\bar{\lambda }}_{R}$ are baseline values in the absence of feedback, *k*_*i*_ are regulation constants, and *m*_*i*_ are Hill exponents. Normalization gives


$$p_{3}(t)=p_{3}(\bar{W}(t)):=1-p_{1}(\bar{W}(t))-p_{2}(\bar{W}(t)).$$

Therefore, *p*_3_ is determined by normalization and therefore inherits feedback through *p*_1_ and *p*_2_.

**Fig 1:**
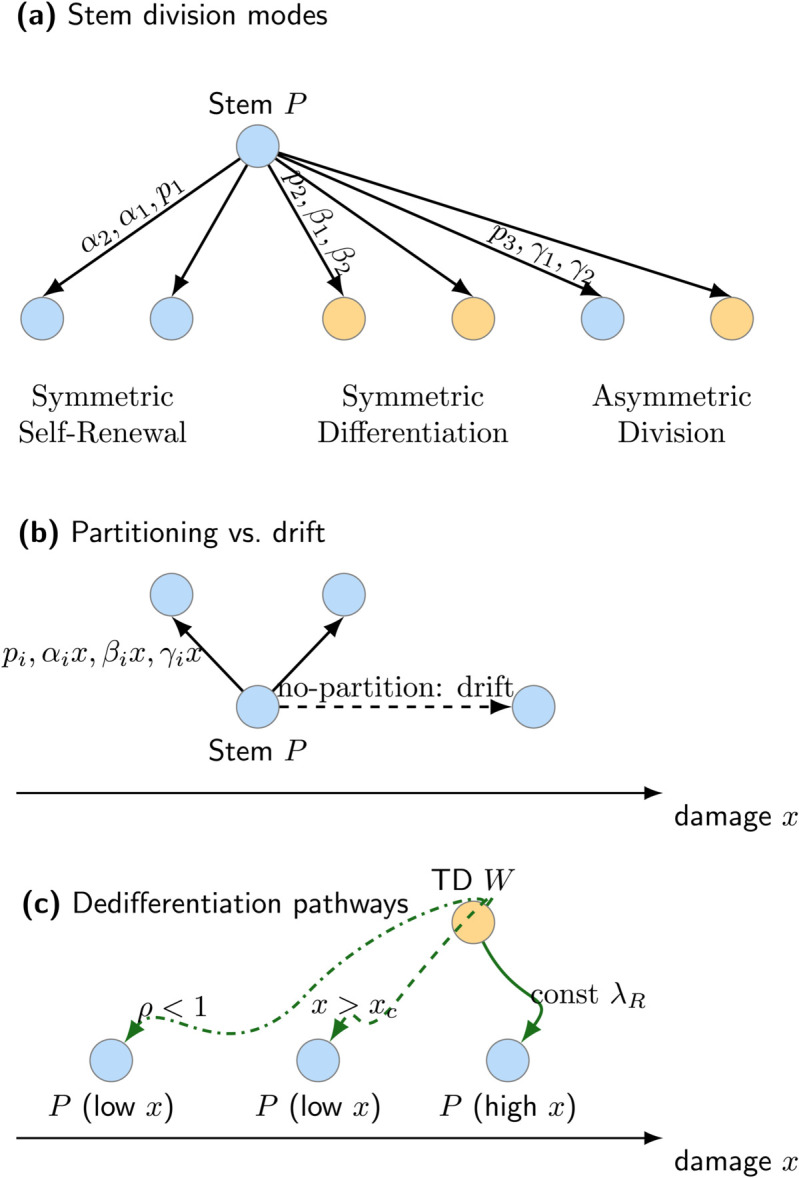
Schematic framework of the damage-structured stem cell lineage model, illustrating division modes, damage inheritance, and dedifferentiation pathways. **(a)** Stem cell undergoes three distinct division types, including symmetric self-renewal, symmetric differentiation, and asymmetric division, with probabilities *p*_1_, *p*_2_, and *p*_3_, respectively. The first type is symmetric self-renewal, which generates two stem daughters. The second type is symmetric differentiation, which generates two TD daughters. The last type is asymmetric division, which generates one stem and one TD daughter. Upon division, damage of the mother cell is distributed to offspring according to partitioning fraction $\alpha_{1,2}$ in symmetric self-renewal, $\beta_{1,2}$ in symmetric differentiation, and $\gamma_{1,2}$ in asymmetric division. **(b)** Comparison of damage evolution scenarios shows that nonlocal partitioning limits damage accumulation, resulting in a bounded stationary distribution. In the absence of partitioning, damage accumulates indefinitely due to constant aging rates $v_{P}$ and $v_{W}$, leading to an unbound rightward drift of the damage distribution. **(c)** Three pathways allow TD cells to revert to the stem state: (i) constant dedifferentiation at a fixed rate $\lambda_{R}$; (ii) threshold-dependent dedifferentiation (TDD), where dedifferentiation occurs only if damage exceeds a critical threshold *x*_*c*_; and (iii) partial repair, where dedifferentiating cells undergo damage repair determined by the retention fraction ρ<1.

As shown in Fig 1, stem cells undergo symmetric self-renewal, symmetric differentiation, or asymmetric division (Fig 1a). Partitioning ensures bounded damage dynamics (Fig 1b), whereas dedifferentiation pathways (Fig 1c) govern how damaged TD cells revert to the stem population.

#### Biological interpretation of the mathematical model.

The terms in Eq (1) capture four biological processes. (i) Aging: both populations accumulate damage at constant rates $v_{P}$ and $v_{W}$. (ii) Replication with partitioning: stem cells divide at a rate $\lambda_{P}$, with the levels of damage to the offspring determined by transition kernels. The three replication events are nonlocal. Transition kernels $K_{1,P}(x,x^{\prime })$ and $K_{3,P}(x,x^{\prime })$ describe how stem offspring with damage level *x* arise from a stem parent with damage *x*^′^ during symmetric self-renewal and asymmetric division. Similarly, $K_{2,W}(x,x^{\prime })$ and $K_{3,W}(x,x^{\prime })$ specify how TD offspring with damage *x* arise from a stem parent with damage *x*^′^ during symmetric maturation and asymmetric division. (iii) Apoptosis: TD cells die at rate δ(x), a nondecreasing function of *x*. Here, we interpret *x* as a normalized measure of cumulative damage, so larger *x* values correspond to proportionally higher death risks. For example, δ(x)=0.6x saturates lethality near x≈1. (iv) Dedifferentiation: TD cells revert to the stem population at a rate $\lambda_{R}$. A crucial modeling assumption is that the dedifferentiation term is local such that the process preserves the damage level *x*. This means that a TD cell with damage level *x* becomes a stem cell with the same damage level *x*.

The dedifferentiation rate $\lambda_{R}$ is modeled in several ways: (i) as a constant parameter $\lambda_{R}$ in the baseline model, (ii) as the baseline constant ${\hat{\lambda }}_{R}$ before regulation, (iii) as a feedback-regulated rate depending on the total stem population,


$$\lambda_{R}=\lambda_{R}(t)=\lambda_{R}(\bar{P}(t)),\;\bar{P}(t):=\int_{0}^{\infty }P(t,x)\;dx,$$

or (iv) a damage-dependent function $\lambda_{R}(x)$ in the TDD setting in Results section. We explicitly indicate in each case whether $\lambda_{R}$ is constant, time-dependent, or damage-dependent. Analogous conventions apply to *p*_1_, *p*_2_, the renewal fraction *f*, the death rate *δ*, and the replication rate $\lambda_{P}$. Feedback is applied on *p*_1_, *p*_2_, $\lambda_{P}$, and $\lambda_{R}$. By contrast, the death rate *δ* is specified as either constant or damage-dependent. The explicit Hill-function forms of these feedback regulations are given in Eqs (8)–(11). For brevity, we sometimes use shorthand such as *p*_1_(*t*) or $\lambda_{P}(t)$ to denote $p_{1}(\bar{W}(t))$ and $\lambda_{P}(\bar{W}(t))$, respectively.

The scaling in Eq (3) guarantees proper distribution of damage and mass conservation across daughter lineages. Replication with nonlocal partitioning recycles stem cells to lower-damage states, opposing the rightward drift from advection. The interplay of these forces produces stationary damage profiles confined within bounded ranges.

The advection terms in Eq (5) shift damage progressively toward larger *x*, producing unbounded mean damage. Thus, equilibrium in $\left(\bar{P},\bar{W}\right)$ does not imply stationarity in the damage distribution. By contrast, the nonlocal partitioning in Eq (2) continuously recycles damage to lower states, yielding bounded stationary profiles once $\left(\bar{P},\bar{W}\right)$ stabilize. Appendix details a two-step control strategy for Eq (6), which facilitates our parameter calibrations to achieve desired steady states in simulations.

Biologically, the boundary conditions in Eq (7) mean that no new stem cells enter with exactly zero damage—all stem cells arise from divisions of already-damaged cells. In transport terms, the boundary condition rules out any artificial influx of pristine cells at the left boundary of the damage domain.

We next give a model extension where damage repair during dedifferentiation is state-dependent.

**Remark 1** (State-dependent repair during dedifferentiation). *Activation of stem cell reprogramming machinery occurs during dedifferentiation. This process can trigger various repair mechanisms. These mechanisms include epigenetic resetting, enhanced proteostasis, or DNA repair. These repairs reduce the amount of damage returning to the stem population. To represent this mechanistically, we propose an extension where we replace the simple deterministic retention fraction ρ by a state-dependent retention function $\rho =\rho (x^{\prime })\in [0,1]$. Concretely, the dedifferentiation kernel*


$$r(x|x^{\prime })=\delta (x-\rho x^{\prime })$$

*is generalized to*


$$r(x|x^{\prime })=\delta (x-\rho (x^{\prime })\;x^{\prime }),$$

*so that a TD cell with damage x*^*′*^ *returns with reduced damage $\rho (x^{\prime })x^{\prime }$. Assume $x^{\prime }\mapsto x^{\prime }\rho (x^{\prime })$ is a one-to-one function in x*^*′*^*. The corresponding nonlocal dedifferentiation terms in the PDE become*

$${𝒟}_{P}[P,W](t,x)\;=\;\lambda_{R}\int_{0}^{\infty }r(x|x^{\prime })\;W(t,x^{\prime })\;dx^{\prime }\;=\;\frac{\lambda_{R}}{\rho (x^{*})}W\;(t,\frac{x}{\rho (x^{*})}).$$ (12)

Here, x^*^ *is the unique number such that $x^{*}\rho (x^{*})=x$. The TD loss from dedifferentiation is the matching nonlocal term*


$${𝒟}_{W}[P,W](t,x)\;=\;-{𝒟}_{P}[P,W](t,x)=-\frac{\lambda_{R}}{\rho (x^{*})}W\;(t,\frac{x}{\rho (x^{*})})$$

*When ρ is constant this reduces to Eq (4).*

*A biologically interpretable parametrization is*


$$\rho (x^{\prime })=\rho_{\min}+(\rho_{\max}-\rho_{\min})\;s(x^{\prime }),\;s(x^{\prime })\in [0,1].$$

*Here, $s(x^{\prime })$ is a monotone function that encodes how reprogramming and repair vary with damage level. For example, $s(x^{\prime })$ could be decreasing so that highly damaged TD cells trigger stronger reprogramming-mediated repair.*

*For analytical tractability, we retained the constant-ρ form in the paper, since it isolates the conservative limit where ρ=1 from the partial-repair case where 0<ρ<1. However, the state-dependent formulation above is straightforward to implement numerically by replacing the algebraic remap by the nonlocal integral $\int r(x|x^{\prime })W(t,x^{\prime })\;dx^{\prime }$. It also allows direct testing of hypotheses in which repair efficiency depends on damage.*

#### Limitation and modeling justification.

Having established the governing PDEs together with boundary conditions and feedback regulations, we now examine the assumptions made when formulating the model. Our PDE system simplifies stem cell hierarchies to highlight the interplay between dedifferentiation and damage partitioning. Assumptions such as fixed transitions and constant drift provide a tractable foundation for studying long-term stability and aging dynamics. More elaborate hierarchies or repair mechanisms could refine quantitative thresholds, but would not alter the qualitative insights we draw.

First, stem cells *P*(*t*,*x*) are modeled as long-lived without explicit attrition or quiescence. While consistent with their relative longevity [26], apoptosis, senescence, or niche exit are well documented [8]. Stem attrition can be incorporated by adding a death term $\delta_{P}(x)$, which increases the replication burden required to offset loss. Quiescence can be represented by an additional compartment *Q*(*t*,*x*) with bidirectional transition rates $s_{P}(x),r_{Q}(x)$, buffering stochastic loss and altering steady-state damage distributions. We explicitly incorporate stem cell attrition and quiescence in the Appendix. In either case, feedback regulation can be re-tuned to maintain homeostasis. The qualitative role of dedifferentiation—as a rescue mechanism versus an aging driver—should persist, but the parameter window for stability would be narrower.

Experimental studies have identified TA progenitor cells with biphasic behavior, i.e., early-phase amplification followed by late-phase extinction [43]. This continuous, gradual differentiation within the stem cell hierarchy has been documented to be involved with genetic and epigenetic changes [44]. However, TA cells are omitted to sharpen the analysis of dedifferentiation and damage within the stem → TD lineage. This two-compartment reduction enables tractable analysis. However, a three-compartment hierarchy from stem to TA to TD could capture how dedifferentiation interacts with TA proliferation and stem dormancy. Because TA cells divide rapidly [45], they may buffer or amplify damage fluxes and alter population ratios, potentially shifting the regimes where dedifferentiation stabilizes versus destabilizes tissue homeostasis. Exploring these effects is an important direction for future work. Nonetheless, the present two-compartment reduction remains valid for isolating the effects of dedifferentiation and partitioning, which are the central focus of this study. We analyze the role of an intermediate TA population and evaluate the robustness of our reduced stem–TD model in representing dedifferentiation dynamics in the Appendix. We assumed symmetric partitioning fractions with $\alpha_{i}=\beta_{i}=1/2$. Asymmetric choices alter higher moments of the damage distribution but preserve total damage. Parameter sweeps in the Results section confirmed their limited impact on long-term damage statistics.

We impose homogeneous Dirichlet boundary conditions with P(t,0)=W(t,0)=0, which preclude inflow of undamaged cells. Because *x* is a continuous damage variable, the singleton x=0 has measure zero and does not affect population integrals. Moreover, positive partition fractions ensure that divisions never produce strictly undamaged progeny, even under highly asymmetric partitioning. Alternative Robin-type inflows can represent a sustained influx of minimally damaged cells [46,47]. Parameter sweeps in the Results section show that damage statistics remain robust under modest inflows.

Damage repair was omitted beyond dilution by partitioning. More general models could introduce an explicit repair mechanism that counteracts accumulation, such as leftward drift or a repair flux. Likewise, dedifferentiation was assumed to preserve damage exactly: a TD cell reverting to the stem population carries its current *x*, encoded by $\lambda_{R}W(t,x)$. This assumption is analytically tractable and conservative. If stability and the proposed control strategy hold under full retention, the same stability and control are expected to persist under partial repair. Including such a repair term in damage accumulation or dedifferentiation would lower effective damage loads and is expected to broaden the parameter regime in which dedifferentiation remains protective. Thus, while quantitative thresholds may shift, the qualitative dual role of dedifferentiation identified in this work should remain valid. More general formulations include retention kernels or TDD. Importantly, our numerical experiments in the Results section demonstrate that these mechanisms can substantially mitigate damage influx, further underscoring the robustness of the current framework.

Finally, feedback was restricted to symmetric divisions, with asymmetric division *p*_3_ as a default stabilizing mechanism to maintain homeostasis [48,49]. This choice reduces parameter complexity but precludes direct regulation of all pathways. An alternative would allow each division probability $p_{i}(\bar{W})$ to depend on TD cell feedback:


$$p_{i}(\bar{W})=\frac{{\hat{p}}_{i}}{1+(k_{i}\bar{W}{)}^{m_{i}}},\;i=1,2,3$$

subject to $p_{1}+p_{2}+p_{3}=1$. While most simulations here set *p*_3_ = 0, incorporating asymmetric division is biologically relevant [50,51].

In the minimal feedback architecture, we regulate *p*_1_, *p*_2_, $\lambda_{P}$, and $\lambda_{R}$ and set $p_{3}=1\;-\;p_{1}\;-\;p_{2}$ by normalization. We do not introduce an additional independent feedback law for *p*_3_ because the constraint $p_{1}\;+\;p_{2}\;+\;p_{3}=1$ makes full independent regulation over-parameterized without additional biological data to identify distinct control pathways. Biologically, asymmetric division is often viewed as a baseline maintenance strategy, while tissue-level signals primarily modulate the renewal–differentiation balance. To verify that this modeling choice does not bias our conclusions, we performed parameter sweeps over *p*_3_ in the Results section. The sweep results show that a moderate *p*_3_ produces transient oscillations but leaves long-term damage statistics largely unchanged.

Another extension of the model that incorporates the feedback regulation of *p*_3_ is the following. We set


$$p_{1}(\bar{W})=\frac{{\hat{p}}_{1}}{1+(k_{1}\bar{W}{)}^{m_{1}}},\;p_{3}(\bar{W})=\frac{{\hat{p}}_{3}}{1+(k_{3}\bar{W}{)}^{m_{3}}},\;p_{2}(\bar{W})=1-p_{1}(\bar{W})-p_{3}(\bar{W}).$$

Then truncate $p_{1},p_{2},p_{3}$ to [0,1] if needed.

The purpose of this subsection is to acknowledge biological simplifications introduced for tractability and to indicate how they could be relaxed in extended models. These structural limitations are distinguished from numerical and methodological limitations, which we revisit in the Discussion after presenting results. With the modeling framework in place, we turn to the numerical discretization and simulation methods used to analyze these equations.

### Numerical methods and verification

This section establishes the computational framework used to analyze our PDE model of stem–TD hierarchies. The objectives are to verify that the discretization is accurate, conservative, and stable. We then apply the scheme in simulations that probe how partitioning, dedifferentiation, and repair shape lineage dynamics. We begin by presenting the finite-volume scheme and its stability properties, then confirm its first-order accuracy with manufactured-solution tests. We next introduce parameter choices that anchor the simulations in biological reality, before turning to baseline and extended scenarios in later sections.

Since the model is deterministic, standard hypothesis testing with p-values is not applicable. Instead, model robustness and validity were evaluated via grid convergence analysis with *R*^2^ values and systematic parameter sensitivity sweeps.

#### Finite-volume upwind scheme.

We first describe the discretization, which provides the foundation for all subsequent simulations. A finite-volume upwind scheme in space, coupled with the forward Euler time-stepping scheme, is used to approximate the PDE system. Nonlocal partitioning terms are computed by linear interpolation, preserving total mass and maintaining second-order spatial accuracy. Because both the upwind discretization and Euler step are first-order, the overall method is first-order accurate, as later confirmed by grid refinement. Stability requires the usual CFL condition

$$\Delta t\leq C\;\frac{\Delta x}{\max\{v_{P},v_{W}\}},\;0<C<1,$$ (13)

which is enforced throughout.

We use *C* = 0.25 throughout. For manufactured-solution tests, we adopt a smaller *C* = 0.005 to suppress secondary numerical artifacts, such as phase error or dispersive oscillations, and to cleanly reveal truncation error. The domain is truncated to [0,*A*], with homogeneous Dirichlet conditions at *x* = 0 and outflow at *x* = *A*. Outflow is implemented using zero ghost values and upwind fluxes $F_{i+1/2}=v_{P}P_{i}$, which yield the discrete divergence $v_{P}(P_{i}\;-\;P_{i\;-\;1})/\Delta x$. The combination of zero ghost values and upwind fluxes ensures that all mass leaving the domain is lost and none enters. These influx and outflux results are consistent with the absence of pristine inflows.

#### Conservation checks.

Two properties make the scheme suitable for biological applications. First, it is conservative: mass is preserved in the absence of source and sink terms, so any changes in total mass arise solely from reaction updates. Second, it is stable and first-order convergent, providing a reliable baseline for simulations. All computations were performed in MATLAB R2025a with Update 1 using only core functionality without additional toolboxes. For reproducibility, pseudocode for the update loop and interpolation of division integrals is provided in the Appendix. We also deposit all code supporting current research findings in the GitHub repository https://github.com/Louis-shuo-wang/PloS_one_stem.git. In addition, we also provide detailed difference equations, interpolation formulas, and truncation-error analysis in the Appendix section. Table 1 lists parameter values and ranges used in the subsequent analyses.

**Table 1:** Model parameters used in simulations. Values are either fixed baselines or ranges explored in sensitivity sweeps. All parameters are nondimensional.

Category	Parameter	Description	Value(s)
*Damage accumulation*	$v_{P},v_{W}$	Damage drift speeds for stem and TD populations	0.02, 0.05, 0.2
*Replication & dedifferentiation*	$\lambda_{P}$	Stem replication rate	0.6785–1.1377
	$\lambda_{R}$	TD dedifferentiation rate	0.01–0.09
*Cell loss*	δ(x)	TD death rate	constant: 0.1–0.5 nonlinear: 0.6*x*
*Division probabilities*	$p_{1},p_{2},p_{3}$	Probabilities of self-renewal, differentiation, and asymmetric division	*p*_1_: 0.05–0.5 *p*_2_: 0.05–0.65 *p*_3_: 0–0.9
*Partitioning fractions*	$\alpha_{1,2}$	Fractions for symmetric self-renewal	(0.5, 0.5)
	$\beta_{1,2}$	Fractions for symmetric differentiation	(0.5, 0.5)
	$\gamma_{1,2}$	Fractions for asymmetric division	(1/3, 2/3)
*Dedifferentiation control*	*x*_*c*_	Threshold for TD → stem dedifferentiation	0.6
*Feedback regulation*	*k*_*i*_	Hill regulation constants	*k*_1_: 0.0019–0.3162 $k_{2}=0.1k_{1}$ $k_{3}=k_{4}=0$ or $k_{3}=0.01,\;k_{4}=0.1k_{1}$
	*m*_*i*_	Hill exponents	2

Non-negativity is enforced after each update. For conservation tests, $p_{1}=p_{2}=0.5$, *p*_3_ = 0, $\lambda_{P}=1$, $\lambda_{R}=0$, δ=0, and $k_{1}=k_{2}=k_{3}=k_{4}=0$, on U=[0,2]. Initial conditions are localized,


$$P(0,x)=W(0,x)=5\chi_{\left[0.2,0.4\right]},\;\bar{P}(0)=\bar{W}(0)=1.$$

**Fig 2:**
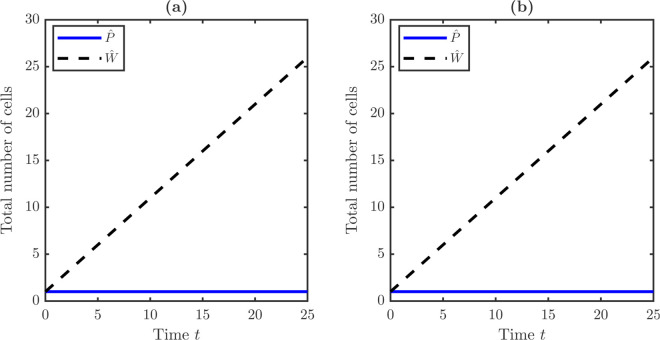
Validation of the numerical scheme: verification of exact mass conservation under damage partitioning. **(a)** Time evolution of total stem and TD cell populations initialized from localized distributions in the absence of drift with $v_{P}=v_{W}=0$. The model assumes conditions of symmetric division and no TD cell loss. These conditions are defined by the parameters δ=0 and $\lambda_{R}=0$. The analytical solution predicts a constant stem cell population. It also predicts a linear increase in the TD cell population. These findings are consistent with the equation $d\bar{W}/dt=\bar{P}=1$. The numerical results confirm these dynamics, demonstrating that the finite-volume scheme preserves mass exactly. **(b)** The introduction of drift, $v_{P}=v_{W}=0.025$, does not violate mass conservation under damage accumulation. The stem total number remains constant, and the TD total number maintains linear growth identical to the no-drift case. This confirms that the conservative finite-volume discretization function correctly without introducing spurious numerical fluxes or instability.

The resulting totals satisfy


$$\frac{d\bar{P}}{dt}=0,\;\frac{d\bar{W}}{dt}=\bar{P}=1,$$

so that stem counts are conserved while TD counts grow linearly. Reaction terms are switched off, and $v_{P},v_{W}$ are varied. Numerical experiments reproduce the exact conservation laws in Fig 2. Fig 2a shows that, in the no-drift case with $v_{P}=v_{W}=0$, stem counts remain constant, and TD counts grow linearly. Fig 2b shows that the same behavior is observed in the drift case with $v_{P}=v_{W}=0.025$. The consistent numerical behaviors across no-drift and drift cases verify conservation and confirm the absence of spurious fluxes.

With the numerical method established, we next verify the accuracy of the discretization through manufactured-solution tests before applying the scheme to biologically motivated simulations.

#### Manufactured-solution convergence tests.

To confirm the expected convergence properties, we next test the scheme against manufactured traveling-wave solutions. We tested the model against manufactured traveling-wave solutions. This test verifies the first-order accuracy of our discretization. These results establish a reliable computational platform. We use this platform to analyze how dedifferentiation influences damage dynamics within the model.

The manufactured traveling-wave profiles are such that the transport operator $\partial_{t}+v\partial_{x}$ vanishes:


$$P(t,x)=2\exp\left(-\frac{(x-(0.5+v_{P}t){)}^{2}}{2\cdot 0.15^{2}}\right),\;W(t,x)=1.6\exp\left(-\frac{(x-(0.7+v_{W}t){)}^{2}}{2\cdot 0.2^{2}}\right),$$

with $v_{P}=v_{W}=0.05$ and parameters $\lambda_{P}=1.1,\;\lambda_{R}=0.07,\;{\hat{p}}_{1}={\hat{p}}_{2}=0.5,\;\delta (x)=0.6x,\;k_{i}=0$ on U=[0,2]. Grid refinement uses Δx∈{0.02,0.01,0.005,0.0025} with Δt∝Δx and the CFL number  = 0.005. Errors are measured in *L*^1^ and $L^{\infty }$ norms for both stem and TD populations at *T* = 2. For these errors, we estimate the observed convergence orders,


$$p\approx \log_{2}\;\left(\frac{E_{\Delta x}}{E_{\Delta x/2}}\right).$$

**Fig 3:**
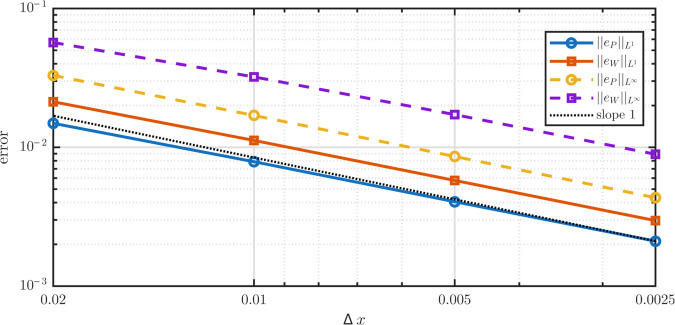
Log–log error versus grid spacing Δx for manufactured traveling-wave solutions of the full PDE. Parameters are $\lambda_{P}=1.1,\lambda_{R}=0.07,{\hat{p}}_{1}={\hat{p}}_{2}=0.5,\delta (x)=0.6x,k_{i}=0,A=2$. Time steps satisfy Δt∝Δx with the CFL number  = 0.005. The black dashed line indicates a reference curve with slope 1 for comparison. Both *L*^1^ and $L^{\infty }$ errors for stem and TD populations decrease with slope ≈1, confirming first-order accuracy of the fully discrete scheme, including conservative remap.

Results are reported in Table 2 and Fig 3. Each halving of Δx reduces error by roughly a factor of two, giving observed orders near one in all norms. High *R*^2^ values confirm log–log linearity, verifying that the scheme is first-order accurate and conservative as designed.

**Table 2:** Discrete *L*^1^ and $L^{\infty }$ errors for *P* and *W* at *T* = 2 under grid refinement, together with estimated convergence orders *p* and regression coefficients *R*^2^. Here *N*_*x*_ denotes the number of grid points and Δt the time step. Errors decrease monotonically with *N*_*x*_, and observed orders cluster near 1 in all norms, consistent with the scheme’s first-order truncation error. Slight deviations from exactly 1 arise from boundary/discretization effects. A conservative CFL number *C* = 0.005 was used here to suppress other artifacts. High *R*^2^ values confirm excellent log–log linearity.

Discrete L^1^ and $L^{\infty }$ errors					
$N_{x}$	Δt	$||e_{P}|{|}_{L^{1}}$	$||e_{W}|{|}_{L^{1}}$	$||e_{P}|{|}_{L^{\infty }}$	$||e_{W}|{|}_{L^{\infty }}$
100	0.002	$1.4891\times 10^{-2}$	$2.1291\times 10^{-2}$	$3.2961\times 10^{-2}$	$5.6884\times 10^{-2}$
200	0.001	$7.8775\times 10^{-3}$	$1.1212\times 10^{-2}$	$1.6973\times 10^{-2}$	$3.2159\times 10^{-2}$
400	0.0005	$4.0574\times 10^{-3}$	$5.7740\times 10^{-3}$	$8.5946\times 10^{-3}$	$1.7208\times 10^{-2}$
800	0.00025	$2.1100\times 10^{-3}$	$2.9654\times 10^{-3}$	$4.3370\times 10^{-3}$	$8.9133\times 10^{-3}$
**Estimated convergence orders p**					
$N_{x}$ (finer)		$p_{L^{1}}(P)$	$p_{L^{1}}(W)$	$p_{L^{\infty }}(P)$	$p_{L^{\infty }}(W)$
200		0.9186	0.9252	0.9576	0.8228
400		0.9572	0.9574	0.9817	0.9021
800		0.9433	0.9614	0.9867	0.9491
**Regression coefficients R**^2^ (fit)					
R^2^		0.9999	0.9999	1.0000	0.9990

The grid refinement test verifies that our upwind finite-volume and forward Euler discretization achieve the expected first-order accuracy in time and space, while preserving conservation. With the discretization validated, we next calibrate model parameters to biologically plausible ranges to ensure simulations reflect realistic stem–TD dynamics.

#### Parameter calibration strategy.

Having verified the numerical scheme, we now specify the parameters so that the simulated dynamics remain biologically grounded. The parameter choices in Table 1 are guided by tissue ratios, division rates, and previous modeling studies, with ranges selected to balance empirical plausibility and numerical stability.

Let $\bar{W}$ and $\bar{P}$ denote the numbers of TD and stem cells, respectively. Parameter values are summarized in Table 1 and are guided by prior modeling studies and biological evidence. TD cells typically outnumber stem cells across tissues. For example, keratinocyte stem cells make up only 4–7% of basal epidermal cells [52]. Stem cells are generally scarce relative to their differentiated progeny. We fix the ratio $\bar{W}/\bar{P}$ at 7 based on this scarcity. This value lies within a biologically reasonable range of 4 to 10, reported by previous work [34]. Damage is assumed to be partitioned symmetrically in self-renewal and differentiation, with $\alpha_{1}=\beta_{1}=0.5$. In asymmetric divisions, replication-induced errors are passed preferentially to differentiation-destined daughters [2], so that stem daughters inherit less damage, modeled by $\gamma_{1}=\frac{1}{3}$.

The stem cell division rate $\lambda_{P}$ plays a central role in determining the TD-to-stem ratio, as established by Proposition 1. We conducted simulations using the damage-dependent death rate δ(x)=0.6x. These tests confirm a monotonic relationship between the ratio and $\lambda_{P}$. We therefore calibrate $\lambda_{P}$ within the range 0.6785 to 1.1377 to keep the ratio close to 7. Damage accumulation rates $v_{P}$ and $v_{W}$ appear as drift terms $v_{P}\partial_{x}P$ and $v_{W}\partial_{x}W$, and interact with nonlocal partitioning rules to produce bounded steady-state profiles. By normalizing the damage domain with *x* = 1 as an approximate upper bound, we select $v_{P},v_{W}\in \{0.02,0.05,0.2\}$ to keep steady-state distributions within this range. The chosen drift rates balance biological plausibility with numerical stability, since smaller drift rates also relax the CFL condition and improve computational efficiency.

Mortality is modeled either by a constant rate δ∈[0,1] or by a linear, damage-dependent death function δ(x)=0.6x. The linear form yields moderate mortality across the biologically relevant range x∈[0,1]. Its effective average, δ≈0.3, is comparable to constant-rate scenarios. The linear function provides a transparent baseline that captures progressive cell fragility and is consistent with observed increases in apoptosis and functional decline as damage accumulates. Empirical data often show exponential rises in dysfunction and cancer incidence with age [53]. We leave nonlinear death functions for future work, since the linear form suffices to isolate the effects of dedifferentiation, feedback, and partitioning.

Division probabilities $\left(p_{1},p_{2},p_{3}\right)$ satisfy $p_{1}\;+\;p_{2}\;+\;p_{3}=1$. A parameter sweep shows that intermediate asymmetric-division probabilities *p*_3_ induce transient overshoots and oscillations in TD-to-stem ratios. Steady-state damage distributions, however, remain largely unaffected; see Results section. To promote resilience and suppress oscillatory behavior, we restrict attention to *p*_3_ = 0 in most simulations, thereby focusing on the balance between symmetric self-renewal and differentiation.

Regulation constants $k_{1},k_{2},k_{3},k_{4}$ scale with steady-state population sizes according to Proposition 1. We impose the constraints $k_{2}=k_{3}=0.1k_{1}$ and $k_{4}=10k_{1}$ to reduce the degrees of freedom in the model. We set $k_{3}=k_{4}=0$ in most simulations, so that the influence of thresholding and repair during dedifferentiation can be examined in isolation. Finally, the Hill exponents $m_{1},m_{2},m_{3},m_{4}$ are fixed at 2, following evidence that lower exponents suppress oscillations, whereas values m≥3 generate unstable steady states in direct simulations [54].

These calibrated choices ensure that simulations remain within physiologically reasonable ranges. With both the numerical scheme and parameters in place, we now proceed to test baseline partitioning, dedifferentiation regimes, and parameter sensitivities.

## Results

We organize the Results to present biology-facing conclusions. We then introduce quantitative metrics used to measure those phenomena, which is followed by dedifferentiation strategies, parameter sweeps, and robustness checks. We present biological insights and their mechanistic interpretations to conclude this section. Mathematical definitions and numerical details are given in Methods.

### Core qualitative biology-facing regimes

We summarize four core, biologically relevant regimes that emerge from the model simulations. Each item states the qualitative outcome and the mechanism driving it. Quantitative evidence is provided in Baseline simulations, Dedifferentiation Scenarios, and Sensitivity sweeps and robustness checks Sections.

#### Partitioning vs. no-partitioning: Homeostasis possible vs. damage runaway.

When intracellular damage is partitioned symmetrically or asymmetrically during stem divisions, the stem population can maintain a low-damage steady-state or homeostasis. By contrast, when partitioning is weak or absent, damage progressively accumulates in the stem population. Consequently, the system enters a damage-runaway regime characterized by steadily increasing mean damage and eventual loss of functional stem capacity. This dichotomy is robust across a wide range of division rates and initial damage distributions.

#### Constant dedifferentiation accelerates stem aging.

A persistent, non-negligible rate of dedifferentiation systematically raises the long-term damage burden in the stem population. In this process, the dedifferentiated TD cells import damage into the stem population, and the polluted stem mother cells segregate damage to the stem and TD daughter cells during division. The eventual result is the aging state in both the stem and TD populations. In simulations with constant dedifferentiation, the stem mean-damage increases faster and attains higher steady-state values than in corresponding no-dedifferentiation controls. The results indicate accelerated stem aging.

#### TD daughters filter damage and protect the stem population.

Dedifferentiation can have a specific threshold. Only TD cells with damage exceeding this threshold can dedifferentiate. These cells then revert to a stem cell state. In this case, dedifferentiation acts as a “detoxification loop." On the one hand, TD cells with high damage possess poor health and fail to perform tissue function effectively. Dedifferentiation allows them to transform into the stem state. They then quickly redifferentiate, go back into the TD population, and are eliminated by the damage-dependent death rate δ(x). On the other hand, this threshold prohibits a large portion of low-damage TD cells from dedifferentiating, protecting the stem population from the damage influx.

#### Partial repair during dedifferentiation mitigates the import of damage.

When dedifferentiation is accompanied by partial repair, the negative impact of dedifferentiation on stem damage is eliminated. To be specific, the damage carried by TD cells during dedifferentiation is reduced to a fraction. Thus, the overall damage imported on the stem population is lesser compared to the constant dedifferentiation case. As a result, damage repair during dedifferentiation is biologically plausible, and many mechanisms, such as epigenetic resetting during reprogramming, confirm our mathematical abstraction.

### Quantification: Metrics and their use

To quantify the regimes above, we introduce a compact set of metrics. These are presented as measurement tools, not as new conceptual layers, and are used consistently across all figures, tables, and parameter sweeps.

Let *P*(*t*,*x*) and *W*(*t*,*x*) denote the stem and TD densities, and $\bar{P}(t)=\int_{0}^{\infty }P(t,x)\;dx$ and $\bar{W}(t)=\int_{0}^{\infty }W(t,x)\;dx$ their total masses.

To quantify the damage distribution, we introduce the compartmental averages.


$$\langle x\rangle_{P}(t)=\frac{\int_{0}^{\infty }xP(t,x)\;dx}{\int_{0}^{\infty }P(t,x)\;dx},\;\langle x\rangle_{W}(t)=\frac{\int_{0}^{\infty }xW(t,x)\;dx}{\int_{0}^{\infty }W(t,x)\;dx}.$$

Compartment averages summarize the central tendency of the damage distribution in each compartment and are the principal metric used to describe stem and TD aging. In addition to compartmental averages $\langle x\rangle_{P}$ and $\langle x\rangle_{W}$, we introduce two complementary metrics to quantify stationary damage profiles. (i) Distribution maxima,


$$P(t,x_{P}^{m}(t))=\max_{x\geq 0}P(t,x),\;W(t,x_{W}^{m}(t))=\max_{x\geq 0}W(t,x),$$

where $x_{P}^{m}$ and $x_{W}^{m}$ denote the damage levels at which stem and TD densities peak. (ii) Rightmost support,


$$x_{P}^{r}(t):=\max\{x\geq 0:P(t,x)>0\},\;x_{W}^{r}(t):=\max\{x\geq 0:W(t,x)>0\},$$

which records the maximal damage present in each population.

To examine the overshoot behavior, we define the overshoot fraction *R* as the relative excess of the transient TD-to-stem ratio $\bar{W}/\bar{P}$ above its steady-state value ${\bar{W}}^{*}/{\bar{P}}^{*}$:


$$R:=\frac{\max\left\{0,\;\left(\max_{t}\frac{\bar{W}(t)}{\bar{P}(t)}\right)-\frac{{\bar{W}}^{*}}{{\bar{P}}^{*}}\right\}}{{\bar{W}}^{*}/{\bar{P}}^{*}},\;\frac{{\bar{W}}^{*}}{{\bar{P}}^{*}}=7.$$

That is, *R* = 0 if the ratio never exceeds its steady-state level, and otherwise *R* measures the maximal percentage overshoot relative to steady state.

To use the above metrics to support the qualitative claims, each numerical experiment is supported by the compartment average, distribution maxima, and rightmost support. They are the main metrics allowing us to conclude the steady state damage distribution, the impact of dedifferentiation mechanisms, and the robustness of the theoretical findings. When we observe overshoot or oscillations in the TD-to-stem ratio dynamics, we use the overshoot fraction to compare the overshoot magnitude.

### Baseline simulations: Partitioning vs. no-partitioning

As a foundation, we compare models with and without partitioning. Partitioning redistributes damage to lower states, enabling bounded stationary profiles; without it, damage drifts unboundedly despite conserved cell counts.

We simulate two systems with identical total population dynamics: (i) the full PDE with nonlocal partitioning in Eq (2), and (ii) a reduced “no-partition” transport model in Eq (5). Parameters are set to ${\hat{p}}_{1}={\hat{p}}_{2}=0.5$, *p*_3_ = 0, ${\hat{\lambda }}_{P}=1$, ${\hat{\lambda }}_{R}=0$, δ=0.5, $v_{P}=v_{W}=0.2$, with initial data $P(0,x)=W(0,x)=10\chi_{\left[2,4\right]}$ on [0,20], yielding a steady state $\left({\bar{P}}^{*},{\bar{W}}^{*})=(20,40\right)$.

**Fig 4:**
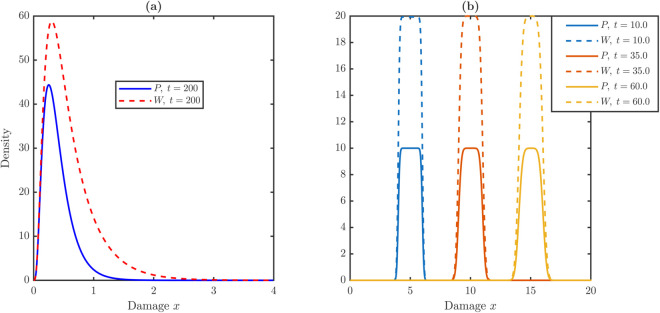
Comparison of damage profile evolution under nonlocal partitioning versus pure advection dynamics. **(a)** Time evolution of stem and TD damage distributions in the full model in Eq 2. Under symmetric division with ${\hat{p}}_{1}={\hat{p}}_{2}=0.5$ and constant damage accumulation with $v_{P}=v_{W}=0.2$, the nonlocal partitioning kernel recycles damage to lower states. This counteracts the advection drift, causing the profiles to rapidly converge to bounded stationary distributions concentrated at low damage levels where x≤1 by *t* = 30. **(b)** Evolution of damage profiles in the absence of partitioning in Eq (5), where division does not redistribute damage. Despite identical total population counts to case (a), the damage distributions drift continuously to the right. The mean damage increases linearly with time, confirming that equilibrium in cell numbers does not guarantee homeostasis in damage levels without partitioning.

Fig 4 illustrates the damage distributions for stem and TD cells with or without damage partitioning. With partitioning, the distributions rapidly converge to stationary bounded profiles concentrated at low damage in Fig 4a. By *t* = 30, the averages stabilize at $\langle x\rangle_{P}=0.4$ and $\langle x\rangle_{W}=0.6$, with densities essentially confined to x≤1. In contrast, the no-partition model exhibits unbounded rightward drift despite identical total counts in Fig 4b. Least-squares fits confirm linear growth of the averages,


$$\langle x\rangle_{P}(t)\approx 0.2000t+2.9900,\;\langle x\rangle_{W}(t)\approx 0.2000t+2.9896.$$

Partitioning leaves overall cell counts unchanged but qualitatively reshapes damage dynamics by preventing runaway accumulation and ensuring bounded steady distributions. Biologically, these results imply that the partition of molecular damage is essential for tissue homeostasis. Without it, the model predicts an inexorable accumulation of damage and stem cell collapse, inconsistent with observed long-term maintenance. Having established that partitioning is essential, we next test whether dedifferentiation rescues or destabilizes the stem population.

### Dedifferentiation scenarios

We now evaluate constant, threshold-dependent, and repair-modulated dedifferentiation. Constant backflow imports TD cells indiscriminately, accelerating stem aging. TDD instead permits only TD cells whose damage exceeds a threshold *x*_*c*_ to revert, reducing the inflow of moderately damaged cells. Partial repair further lightens the burden by diminishing the damage carried during reversion. These distinct mechanisms allow us to test how regulation shapes damage distribution profiles for stem and TD populations.

#### Constant vs. TDD.

A constant rate of dedifferentiation $\lambda_{R}$ indiscriminately feeds TD cells back into the stem population, potentially importing damaged cells and accelerating stem aging. In contrast, experimental studies suggest that dedifferentiation is not uniform and can be triggered only when damage exceeds a threshold [16,55]. Therefore, TDD activates only when *x*>*x*_*c*_:


$$\lambda_{R}(x)=\left\{ \begin{array}{cc} 0, & 0\leq x<x_{c},\\ \text{constant}, & x\geq x_{c}. \end{array}\right.$$

TD cells are diverted from highly damaged cells back into the stem population. TDD could act as a detoxification loop, selectively protecting low-damage stem populations.

Simulations are performed on U=[0,2] with


$${\hat{p}}_{2}=1-{\hat{p}}_{1},\;p_{3}=0,\;{\hat{\lambda }}_{R}\in \{0.01,0.03,0.05,0.07,0.09\},\;\delta =0.6x,\;k_{2}=0.1k_{1},\;k_{3}=k_{4}=0,\;v_{P}=v_{W}=0.05,\;x_{c}=0.6.$$

and initial condition $P(0,x)=W(0,x)=10\chi_{\left[0.2,0.4\right]}$. To preserve the consistent steady-state ratio of 7, the parameters ${\hat{p}}_{1},{\hat{p}}_{2},{\hat{\lambda }}_{P},k_{1}$ are calibrated according to the scaling law in Proposition 1.

**Fig 5:**
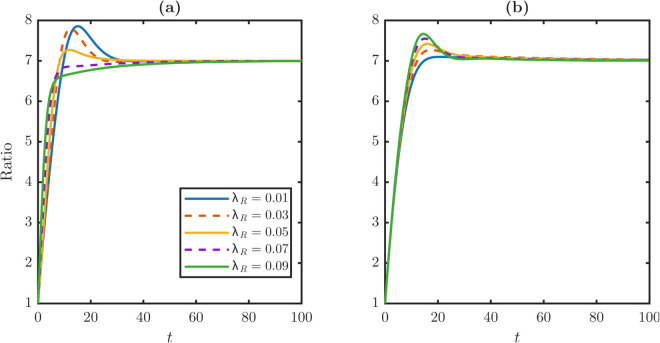
Temporal evolution of the population ratio $\bar{W}/\bar{P}$ under different dedifferentiation rules. **(a)** The system ratio exhibits overshoot before converging to the stable steady-state value under constant dedifferentiation rate. Higher dedifferentiation rates reduce overshoot magnitude and even eliminate overshoot when $\lambda_{R}\geq 0.07$. **(b)** Dynamics under a threshold-dependent rule where only cells with damage over *x*_*c*_ can dedifferentiate. The system ratio still demonstrates overshoot before stabilizing at the steady-state value. Unlike the constant dedifferentiation case, larger dedifferentiation produces a transient overshoot. This overshoot arises because high-damage TD cells must first accumulate more damage, which must reach the threshold *x*_*c*_. The cells then revert to the stem population, and this requirement creates a time delay. This delay temporarily inflates the TD population relative to the stem cell count.

Under dynamic conditions, we track both population ratios and damage distributions. Since TD cells vastly outnumber stem cells [56], with reported TD-to-stem ratios of 4–10 [34], we fix the control ratio at 7 to anchor simulations. Both constant dedifferentiation and TDD converge to the stable TD-to-stem ratio of 7, but by different routes. Fig 5 shows the ratio dynamics under constant and TDD. Under constant dedifferentiation in Fig 5a, larger $\lambda_{R}$ values yield smoother convergence. Under TDD in Fig 5b, however, they produce a transient overshoot, reflecting the delayed reentry of damaged TD cells after crossing the threshold *x*_*c*_.

**Fig 6:**
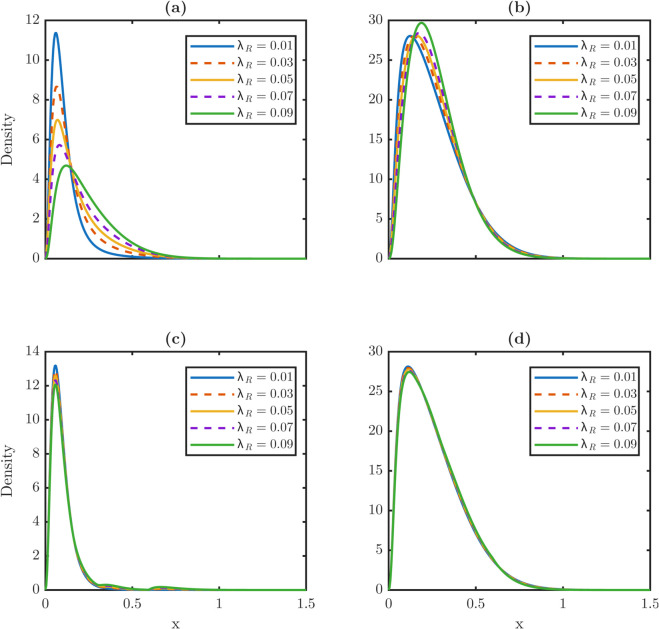
Impact of dedifferentiation strategies on steady-state damage distributions for stem and TD populations: constant vs. TDD. **(a-b)** Steady-state damage profiles for stem and TD populations under a constant dedifferentiation rate. In this regime, dedifferentiation indiscriminately recycles cells from the TD population regardless of their damage level. This unrestricted flux of TD damage to the stem population shifts the stem (a) and TD (b) damage distributions toward higher damage states with heavier tails. **(c-d)** Profiles under a threshold-dependent rule where only TD cells with $x\geq x_{c}$ dedifferentiate. TDD restricts the reentry flux to only high-damage cells, preventing the aging of the stem population observed in the constant case. Both stem (c) and TD (d) cell populations effectively mitigate the aging effect induced by dedifferentiation. The mitigation ensures that the distribution maximum remains almost unchanged under different dedifferentiation rates. This mitigating effect maintains a youthful and low-damage distribution of cell populations. Distributions are shown for $\lambda_{R}\in \{0.01,\ldots ,0.09\}$ with $v_{P}=v_{W}=0.05$ and δ(x)=0.6x. The dedifferentiation threshold is set at *x*_*c*_ = 0.6.

Fig 6 contrasts steady-state distributions under constant dedifferentiation and TDD. Under constant dedifferentiation in Fig 6a and 6b, increasing $\lambda_{R}$ shifts $x_{P}^{m}$ and $x_{W}^{m}$ to the right, especially for stem cells, indicating an aging effect. The stem distribution also develops a heavier right tail as $\lambda_{R}$ grows. In contrast, under TDD in Fig 6c and 6d, maxima remain nearly fixed across $\lambda_{R}$, and stem distributions stay centered at low damage levels.

Table 3 quantifies these trends. Under constant dedifferentiation, stem averages nearly double from 0.1150 to 0.2385 and $x_{P}^{m}$ increases twofold from 0.0650 to 0.1250. By contrast, TD averages change only slightly from 0.2601 to 0.2709. The rightmost supports move in opposite directions: $x_{P}^{r}$ expands, whereas $x_{W}^{r}$ contracts. Under TDD, averages and maxima vary minimally, e.g., $x_{P}^{m}$ from 0.0600 to 0.0650, but the divergent shift in supports persists, with $x_{P}^{r}$ increasing and $x_{W}^{r}$ decreasing by comparable magnitudes.

**Table 3:** Metrics of *P* and *W* at steady state under constant dedifferentiation and under TDD with *x*_*c*_ = 0.6. Increasing $\lambda_{R}$ under constant dedifferentiation strongly elevates stem averages and maxima, modestly affects TD metrics, and extends the stem right tail. Under TDD, stem averages vary weakly; TD metrics remain nearly constant. Supports diverge moderately with $x_{P}^{r}$ expanding and $x_{W}^{r}$ contracting in two scenarios.

Regime	$\lambda_{R}$	Stem metrics			TD metrics		
		$\langle x\rangle_{P}$	$x_{P}^{m}$	$x_{P}^{r}$	$\langle x\rangle_{W}$	$x_{W}^{m}$	$x_{W}^{r}$
*Constant*	0.01	0.1150	0.0650	1.2250	0.2601	0.1300	1.4650
	0.03	0.1518	0.0700	1.2850	0.2630	0.1500	1.4400
	0.05	0.1800	0.0750	1.3050	0.2653	0.1650	1.4250
	0.07	0.2064	0.0850	1.3100	0.2676	0.1750	1.4050
	0.09	0.2385	0.1250	1.3050	0.2709	0.1950	1.3850
*TDD (x_c_ = 0.6)*	0.01	0.0958	0.0600	1.2300	0.2589	0.1150	1.4650
	0.03	0.1023	0.0600	1.2950	0.2594	0.1200	1.4450
	0.05	0.1085	0.0650	1.3150	0.2600	0.1200	1.4300
	0.07	0.1142	0.0650	1.3250	0.2605	0.1200	1.4150
	0.09	0.1196	0.0650	1.3250	0.2611	0.1250	1.3950

Together, these results show that constant dedifferentiation accelerates damage accumulation in stem cells but only modestly affects TD cells. The accelerated damage accumulation in stem cells produces an aging phenotype marked by sharply elevated stem averages. TDD suppresses the aging-inducing effect by stabilizing both averages and maxima, though the divergence in the rightmost supports remains. Thus, stem populations are intrinsically more sensitive to dedifferentiation perturbations, and threshold regulation provides a robust protective mechanism against dedifferentiation-induced aging. Since thresholds control which TD cells re-enter the stem population, we next test how partial repair alters damage burden and decouple dedifferentiation from the damage import.

#### Partial-repair dedifferentiation.

In many biological contexts, dedifferentiation is not a perfect reversal of TD-to-stem transition: cells may repair a fraction of accumulated damage during the process. We now numerically explore the generalized kernel introduced in Eq (4) with ρ<1.

The parameters are set as ${\hat{p}}_{2}=1\;-\;{\hat{p}}_{1},\;p_{3}=0,\;{\hat{\lambda }}_{R}\in \{0.01,0.03,0.05,0.07,0.09\},\;\delta =0.6x,\;k_{2}=0.1\;k_{1},\;k_{3}=k_{4}=0,\;v_{P}=v_{W}=0.05$ in U=[0,2] with initial data $P(0,x)=W(0,x)=10\chi_{\left[0.2,0.4\right]}$. To preserve the consistent steady-state ratio of 7, the parameters ${\hat{p}}_{1},{\hat{p}}_{2},{\hat{\lambda }}_{P},k_{1}$ are calibrated according to the scaling law in Proposition 1.

**Fig 7:**
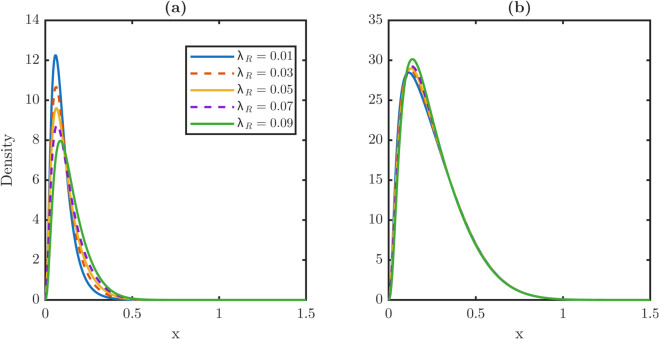
Mitigation of dedifferentiation-induced aging effect through partial repair mechanisms. **(a-b)** Steady-state damage distribution profiles for stem (a) and TD (b) cell populations under a partial repair regime. In this regime, dedifferentiated cells retain only 50% of their accumulated damage with ρ=0.5 in $r(x|x^{\prime })=\delta (x-\rho x^{\prime })$ before they revert to stem cells. Under the constant dedifferentiation setting in Fig 6, high dedifferentiation rates introduce excessive damage into the stem cell population and effectively induce aging in both cell populations. By contrast, the partial repair mechanism acts as a filter, mitigating the damage flux from each TD cell to the stem population. As the dedifferentiation rate increases, the distribution maxima of stem and TD populations remain pinned to low damage values and have negligible rightward shift. The right tail of the stem damage distribution widens while that of the TD damage distribution remains unchanged. This mitigation mechanism shows that even moderate repair capacity is sufficient to neutralize the aging cost associated with high lineage plasticity.

Steady state damage distribution for stem and TD cells under repair-modulated dedifferentiation is demonstrated in Fig 7. Fig 7a shows the stationary stem damage distribution under partial repair, while Fig 7b shows the corresponding TD distribution. Compared to the full retention model, both distributions shift only slightly to the right as $\lambda_{R}$ increases, confirming that repair mitigates the dedifferentiation-induced aging effect. In particular, the stem and TD maxima ($x_{P}^{m}$, $x_{W}^{m}$) remain nearly constant, while the rightmost support of the stem $x_{P}^{r}$ compresses, producing shorter right tails.

Table 4 reports stationary metrics under partial repair. As $\lambda_{R}$ increases, the averages and maxima grow only mildly with $\langle x\rangle_{P}$ rising by less than 50% and $x_{P}^{m}$ by less than 40%. Averages and maxima remain well below the full retention values, which almost double. TD metrics are essentially invariant, with stable means, maxima, and rightmost support $x_{W}^{r}\approx 1.5$. These results show that partial repair interrupts damage transmission from TD to stem cells, thus weakening dedifferentiation-driven aging.

**Table 4:** Summary of steady metrics under ρ = 0.5. Stem average and maxima rise only moderately with $\lambda_{R}$, well below full-retention levels; TD metrics remain essentially invariant.

$\lambda_{R}$	Stem metrics			TD metrics		
	$\langle x\rangle_{P}$	$x_{P}^{m}$	$x_{P}^{r}$	$\langle x\rangle_{W}$	$x_{W}^{m}$	$x_{W}^{r}$
0.01	0.1000	0.0650	0.7450	0.2588	0.1200	1.4750
0.03	0.1126	0.0650	0.7900	0.2594	0.1250	1.4750
0.05	0.1220	0.0700	0.8150	0.2599	0.1350	1.4750
0.07	0.1316	0.0750	0.8300	0.2604	0.1350	1.4750
0.09	0.1471	0.0900	0.8400	0.2614	0.1450	1.4700

Repair during dedifferentiation prevents the stem population from accumulating damage rapidly. Even moderate repair fractions substantially mitigate the effects of aging, stabilizing tissue homeostasis. Unlike constant full-retention dedifferentiation, partial repair decouples flux from heavy damage carry-over. Partial repair also produces stem distributions with lower averages, smaller maxima, and shorter right tails, while leaving TD statistics nearly unchanged. Biologically, the protective effect of partial repair reflects evidence that reprogramming and dedifferentiation often trigger epigenetic remodeling, repair pathways, or metabolic resets. These processes suggest that dedifferentiation coupled with repair may protect the stem population by recycling cells without importing excess damage. Because threshold and repair strongly shape damage distribution, we next vary these control parameters *x*_*c*_ and *ρ* systematically.

### Sensitivity sweeps and robustness checks

To assess robustness, we perform sensitivity sweeps to verify the robustness of the damage-mitigating effects of the two dedifferentiation mechanisms: TDD and partial repair. In addition, we sweep division probabilities, boundary inflows, and partitioning asymmetries. These additional simulations extend the previous subsection by probing sensitivities of parameters that shape stem–TD dynamics. Varying the division probability shifts the balance between stem self-renewal and differentiation, altering the effective supply of cells available for dedifferentiation. Adjusting boundary inflow conditions further tests robustness by controlling the replenishment of low-damage states. Finally, partitioning asymmetry induces transient overshoots in damage distributions, reflecting short-term instability from uneven inheritance. Long-term boundedness, however, is preserved. Taken together, these parameter sweeps demonstrate that the qualitative behaviors identified earlier persist across a broad range of biological assumptions. The findings are therefore robust and not sensitive to specific parameter choices.

#### Threshold sweep: Effect of varying *x*_*c*_ on overshoot and damage filtering.

We vary the threshold parameter *x*_*c*_ that triggers the dedifferentiation in the TDD rule. Biologically, *x*_*c*_ determines how damaged a TD cell must be before reentry into the stem population. Too low a threshold risks importing moderate-damage cells, while too high a threshold delays detoxification feedback.

**Fig 8:**
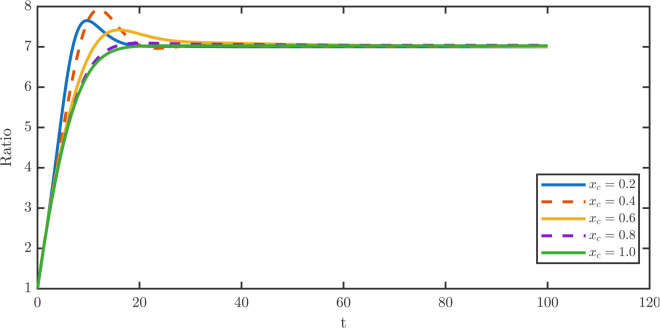
Non-monotone transient dynamics of the TD-to-stem ratio $\bar{W}/\bar{P}$ under varying dedifferentiation thresholds $x_{c}\in \{0.2,0.4,0.6,0.8,1.0\}$ at fixed $\lambda_{R}=0.05$. The system ratio exhibits a transient overshoot before stabilizing at the steady-state value. In the low threshold regime where *x*_*c*_ = 0.2, the population ratio behaves similarly to the constant dedifferentiation case, showing transient overshoot before converging to the stable steady-state value. In the intermediate regime where *x*_*c*_ = 0.4, the overshoot magnitude peaks, reflecting a pronounced accumulation of TD cells before the stabilization of the population ratio. In the high regime where $x_{c}\geq 0.6$, the overshoot magnitude gradually diminishes and nearly disappears when *x*_*c*_ = 1.0. At this extreme value, the threshold approaches the effective maximum damage limit where death dominates, rendering the dedifferentiation flux negligible.

**Fig 9:**
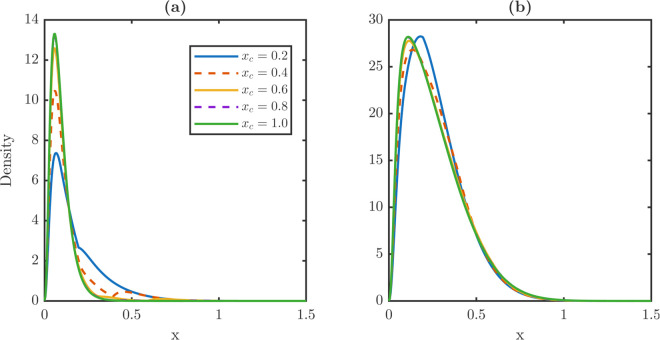
Sensitivity of steady-state damage distributions to the dedifferentiation threshold $x_{c}\in \{0.2,0.4,0.6,0.8,1.0\}$ at fixed $\lambda_{R}=0.05$: the transition from aging to detoxification. **(a)** For the steady-state damage profiles for the stem population across varying thresholds, the location of the distribution maxima remains nearly coincident for all *x*_*c*_, indicating that the stem population’s damage-partitioning mechanism effectively buffers the impact of varying re-entry fluxes. **(b)** The steady-state profiles for the TD population show moderate sensitivity to *x*_*c*_. A low threshold (*x*_*c*_ = 0.2) mimics indiscriminate constant dedifferentiation, coupling dedifferentiation with aging, and induces a rightward shift of TD distribution maxima. As the threshold increases, the TDD mechanism progressively mitigates the aging effect, shifting the TD distribution maxima slightly leftward and demonstrating the protective detoxification loop function.

Fig 5 shows that dedifferentiation suppresses overshoot at constant rates but amplifies it under TDD. Fig 8 shows that increasing *x*_*c*_ delays the overshoot, while its magnitude peaks at intermediate *x*_*c*_. Fig 9 demonstrates the steady-state damage distributions for stem and TD cells across *x*_*c*_ sweeps. Fig 9a illustrates the stationary stem damage distributions, whose maxima remain aligned across thresholds. Fig 9b shows the corresponding TD distributions. At *x*_*c*_ = 0.2, TDD exacerbates aging via a rightward shift, whereas for $x_{c}\geq 0.4$ thresholding progressively mitigates the aging effect by shifting the TD maxima left.

Quantitative comparisons in Table 5 reveal three strong dependencies on *x*_*c*_. Stem mean damage decreases with $\langle x\rangle_{P}:0.1799\to 0.0928$, TD maxima shift left with $x_{W}^{m}:0.1850\to 0.1150$, and the stem right tail extends with $x_{P}^{r}:0.1350\to 0.3350$. Other metrics vary little. Overshoot peaks at *x*_*c*_ = 0.4 with *R* = 0.1317 and nearly vanishes at *x*_*c*_ = 1 with *R* = 0.0039.

**Table 5:** Steady damage metrics for stem and TD populations and overshoot fraction *R* under TDD. The stem average decreases with *x*_*c*_, while TD maxima shift left. The overshoot fraction *R* attains its maximum at *x*_*c*_ = 0.4 and its minimum at *x*_*c*_ = 1.0.

$x_{c}$	Stem metrics			TD metrics			*R*
	$\langle x\rangle_{P}$	$x_{P}^{m}$	$x_{P}^{r}$	$\langle x\rangle_{W}$	$x_{W}^{m}$	$x_{W}^{r}$	
0.2	0.1799	0.0700	1.3050	0.2657	0.1850	1.4200	0.0931
0.4	0.1425	0.0650	1.3050	0.2630	0.1400	1.4250	**0.1317**
0.6	0.1085	0.0650	1.3150	0.2600	0.1200	1.4300	0.0599
0.8	0.0955	0.0600	0.3250	0.2589	0.1150	1.4400	0.0135
1.0	0.0928	0.0600	0.3350	0.2588	0.1150	1.4500	**0.0039**

Overall, the sweep reveals a trade-off: small *x*_*c*_ values enable rapid dedifferentiation of TD cells, replenishing the stem population and dampening overshoot. At intermediate thresholds, dedifferentiation activates only after TD damage has accumulated, amplifying transients. At high thresholds, heavily damaged TD cells tend to die, suppressing overshoot. Therefore, TDD emerges as a protective mechanism: by restricting dedifferentiation to older TD cells, the process both reduces long-term stem aging and limits harmful transient overshoots.

#### Repair fraction sweep: Effect of varying *ρ.*

We next examine partial-repair dedifferentiation, where a TD cell returning to the stem population repairs only a fraction of its accumulated damage. The repair fraction models reprogramming or metabolic resetting that accompany dedifferentiation.

**Fig 10:**
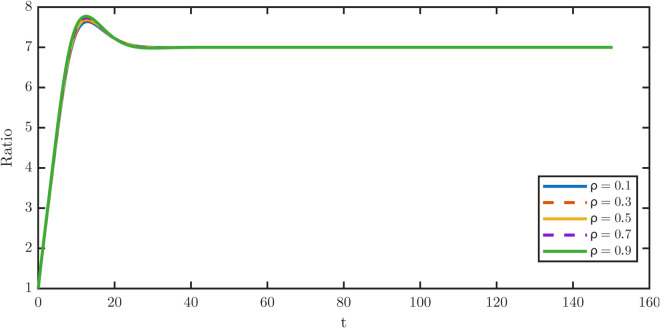
Sensitivity of the TD-to-stem ratio $\bar{W}/\bar{P}$ to retention fractions ρ∈ {0.1,0.3,0.5,0.7,0.9} during dedifferentiation. The magnitude of the transient overshoot grows steadily as repair efficiency decreases, increasing monotonically from 0.0913 at ρ=0.1 to 0.1103 at ρ=0.9 in Table 6. The repair mechanism during dedifferentiation induces a transient accumulation of TD cells. By contrast, the indistinguishable ratio curves across ρ∈{0.1,0.3,0.5,0.7,0.9} indicate negligible sensitivity of the ratio to damage retention fraction *ρ*.

Fig 10 shows the overshoot fraction *R* as a function of *ρ*. The overshoot remains modest throughout the sweep but grows monotonically with *ρ*, rising from 0.0913 at ρ=0.1 to a maximum of 0.1103 at ρ=0.9. Thus, greater retention of TD damage exacerbates the transient imbalance between the TD and stem populations. By contrast, repair with ρ<1 alleviates the overshoot by reducing the damage carried by dedifferentiated TD cells.

**Fig 11:**
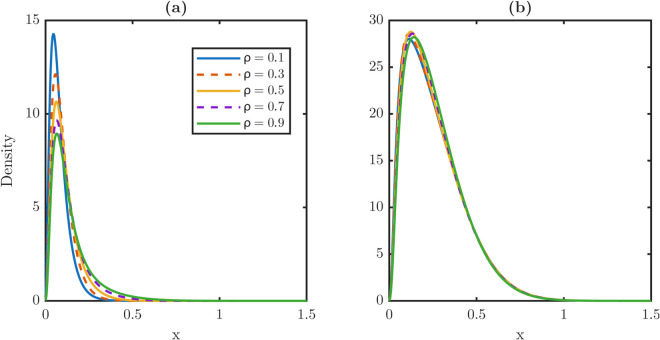
Sensitivity of steady-state damage distributions for stem and TD populations to damage retention fraction ρ. **(a)** Steady-state damage distribution profiles for the stem population as a function of the retention fraction *ρ*. The distribution shifts progressively but slightly toward higher damage values as *ρ* increases from 0.1 to 0.9. This indicates that the stem population shows minor sensitivity to damage repair. **(b)** Corresponding damage profiles for the TD population. Unlike the stem population, the TD distribution remains nearly invariant across all values of *ρ*. This guarantees that the steady-state TD profile is primarily governed by its own internal dynamics rather than the damage repair mechanism. The sensitivity discrepancy between stem and TD populations indicates that repair primarily affects stem quality while leaving TD relatively unaffected.

The corresponding steady-state distributions are plotted in Fig 11. Fig 11a shows the stem-cell distributions, which shift progressively rightward as *ρ* increases, while Fig 11b shows the TD distributions, which remain nearly invariant. These results confirm that repair affects stem quality more strongly than TD quality.

Table 6 quantifies these effects. The stem average $\langle x\rangle_{P}$ rises monotonically from 0.0811 at ρ=0.1 to 0.1439 at ρ=0.9, which shows a nearly 80% increase, while the TD average remains essentially constant between 0.2583–0.2622. Similarly, the stem maxima $x_{P}^{m}$ shift from 0.050 to 0.070, and the rightmost support $x_{P}^{r}$ nearly doubles from 0.6650 to 1.1900, whereas the TD metrics change only slightly. These numerical contrasts highlight that partial repair specifically protects the stem population from damage accumulation, without strongly perturbing the TD population.

**Table 6:** Steady damage metrics for the stem and TD populations across *ρ.* Stem average rises monotonically with ≈80% increase across the sweep, while the TD average remains nearly constant. Overshoot fraction *R* increases with *ρ*, attaining its maximum at ρ=0.9.

ρ	Stem metrics			TD metrics			*R*
	$\langle x\rangle_{P}$	$x_{P}^{m}$	$x_{P}^{r}$	$\langle x\rangle_{W}$	$x_{W}^{m}$	$x_{W}^{r}$	
0.1	0.0811	0.0500	0.6650	0.2583	0.1150	1.4750	0.0913
0.3	0.0969	0.0600	0.6850	0.2585	0.1150	1.4750	0.0948
0.5	0.1126	0.0650	0.7900	0.2594	0.1250	1.4750	0.0995
0.7	0.1283	0.0700	0.9800	0.2607	0.1400	1.4700	0.1047
0.9	0.1439	0.0700	1.1900	0.2622	0.1450	1.4600	**0.1103**

The repair-fraction sweep shows that even modest repair substantially protects the stem population from dedifferentiation-induced damage. Stem averages, maxima, and right tails all decrease under partial repair. Biological evidence supports this mechanism, as dedifferentiation is often accompanied by epigenetic remodeling or DNA repair. Such coupling enables safe recycling of cells without importing excessive damage into the stem population.

Having established these regulatory effects, we now test whether the conclusions hold under broader parameter changes, including division probabilities, boundary inflows, and partitioning asymmetry.

#### Probability sweep: Oscillations and overshoot.

In the baseline model, stem divisions were limited to symmetric self-renewal *p*_1_ and differentiation *p*_2_, with *p*_3_ = 0. We now vary *p*_3_ from 0.1 to 0.9 while maintaining $p_{1}+p_{2}+p_{3}=1$ such that $p_{1}=p_{2}=(1-p_{3})/2$.

**Fig 12:**
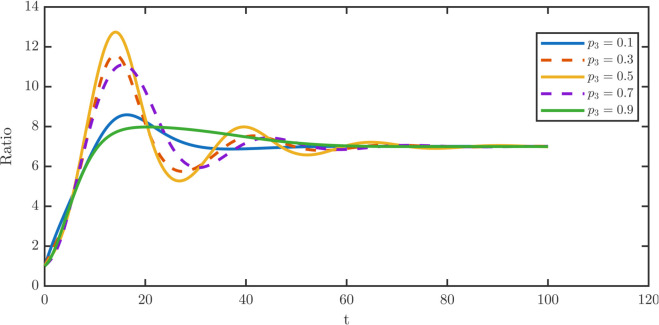
Transient dynamics of the TD-to-stem cell population ratio under varying asymmetric division probabilities *p*_3_. Temporal trajectories of the TD-to-stem ratio $\bar{W}/\bar{P}$ are shown for a range of asymmetric division probabilities $p_{3}\in \{0.1,0.3,0.5,0.7,0.9\}$. To isolate the effect of division asymmetry, simulations assume balanced baseline symmetric renewal and differentiation rates defined by ${\hat{p}}_{1}={\hat{p}}_{2}=(1\;-\;p_{3})/2$, with dedifferentiation inactive such that $\lambda_{R}=0$. The system exhibits transient overshoots and oscillations that behave non-monotonically with respect to *p*_3_. Dynamic instability peaks at intermediate values of asymmetry where *p*_3_ = 0.5 and declines at a high value where *p*_3_ = 0.9. These behaviors indicate that strong asymmetric partitioning can dampen population fluctuations.

Fig 12 shows the temporal ratio dynamics as *p*_3_ increases. Allowing asymmetric division introduces oscillatory behavior not present when *p*_3_ = 0. Both overshoot and oscillation amplitudes display a non-monotone dependence on *p*_3_. They rise with increasing *p*_3_, reach a maximum at intermediate values, and then decline. Larger *p*_3_ also prolongs the duration of the oscillatory phase.

**Fig 13:**
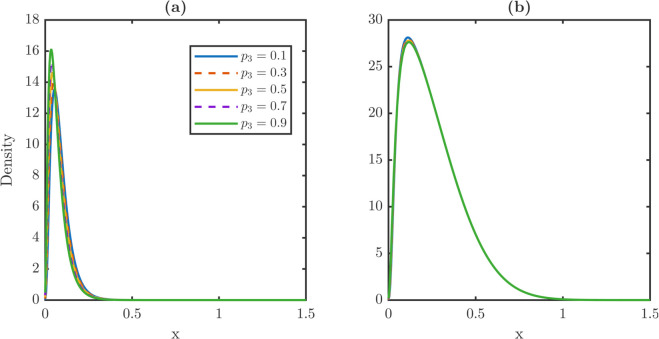
Robustness of steady-state damage distributions to variations in division asymmetry $p_{3}$. The stationary damage density profiles are plotted against the damage variable *x* for increasing asymmetric division probabilities *p*_3_ to assess the sensitivity of lineage quality to partitioning strategies. **(a)** For the stem population, the damage distributions remain highly robust to changes in *p*_3_, exhibiting only a marginal reduction in the distribution maxima as the probability of asymmetric division increases. **(b)** For the TD population, the distributions display a negligible rightward shift in the distribution maxima. The results indicate that the TD damage profile has limited sensitivity to *p*_3_ and that asymmetric division is a secondary factor in damage control in both stem and TD populations.

The corresponding steady-state damage profiles are shown in Fig 13. Fig 13a shows that stem distributions remain robust to changes in *p*_3_, while Fig 13b shows that TD distributions also shift only slightly. In particular, higher *p*_3_ modestly mitigates aging in both populations.

Table 7 quantifies these effects. Stem average $\langle x\rangle_{P}$, maxima $x_{P}^{m}$, and rightmost support $x_{P}^{r}$ all slightly decrease as *p*_3_ increases. The TD average $\langle x\rangle_{W}$ also declines marginally, while the TD maxima $x_{W}^{m}$ shifts modestly rightward and $x_{W}^{r}$ remains invariant. By contrast, the overshoot fraction *R* is highly sensitive: the value peaks at *R* = 0.8198 for *p*_3_ = 0.5 and declines to *R* = 0.1396 at *p*_3_ = 0.9.

**Table 7:** Steady metrics for *P* and *W* and overshoot fraction *R* across *p*_3_. Damage statistics vary modestly, whereas *R* changes strongly: the value peaks near *p*_3_ = 0.5 and is minimized at *p*_3_ = 0.9.

$p_{3}$	Stem metrics			TD metrics			*R*
	$\langle x\rangle_{P}$	$x_{P}^{m}$	$x_{P}^{r}$	$\langle x\rangle_{W}$	$x_{W}^{m}$	$x_{W}^{r}$	
0.1	0.0892	0.0600	0.6650	0.2583	0.1150	1.4750	0.2270
0.3	0.0838	0.0550	0.6600	0.2582	0.1150	1.4750	0.6557
0.5	0.0790	0.0450	0.6550	0.2581	0.1200	1.4750	**0.8198**
0.7	0.0747	0.0450	0.6450	0.2580	0.1250	1.4750	0.5853
0.9	0.0708	0.0400	0.6400	0.2580	0.1250	1.4750	**0.1396**

Biologically, asymmetric division induces transient fluctuations in lineage balance but does not destabilize long-term tissue homeostasis. Since these oscillations leave damage distributions unaffected, this robustness allows us to fix *p*_3_ = 0 in most simulations to avoid transient oscillations and focus on dedifferentiation and feedback. Because steady damage metrics are insensitive to *p*_3_, we keep *p*_3_ unregulated in the baseline model without loss of generality for the damage-focused conclusions.

#### Boundary sweep: Robustness to inflows.

The baseline simulations imposed homogeneous Dirichlet boundaries, excluding inflow of undamaged cells. To test robustness, we relax this assumption and introduce a Robin-type boundary at *x* = 0 that injects minimally damaged cells at rate *g*:


P(t,0)=W(t,0)=g.

Biologically, a constant inflow *g* models continuous recruitment of new, nearly pristine cells from outside the modeled hierarchy. We sweep across the initial and boundary conditions:


$$P(0,x)=W(0,x)=g\chi_{\left[0,2/g\right]},\;P(t,0)=W(t,0)=g,\;g\in \{5,4,3,2,1.5\}.$$

**Fig 14:**
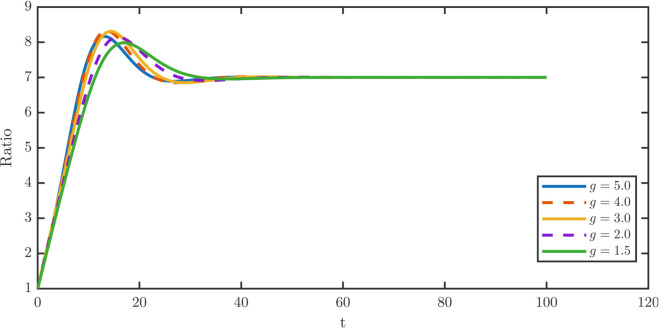
Impact of inhomogeneous Dirichlet boundary conditions on the transient dynamics of the TD-to-stem cell ratio. The temporal evolution of the population ratio $\bar{W}/\bar{P}$ is analyzed under varying inhomogeneous Dirichlet boundary conditions. These boundary conditions are defined by P(t,0)=W(t,0)=g, with inflow values g∈{5,4,3,2,1.5}. These conditions simulate different influxes at the boundary *x* = 0. All tested boundary conditions result in comparable transient dynamics characterized by overshoots. The magnitude of this transient instability peaks at intermediate values of *g*. The peak suggests that moderate boundary inhomogeneities create the largest temporary perturbation in the lineage balance before the system settles into the steady state.

**Fig 15:**
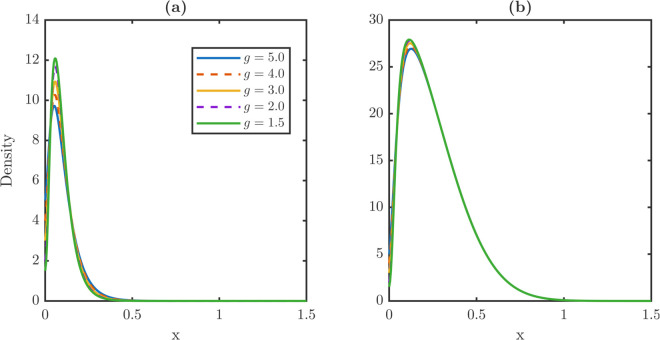
Sensitivity of steady-state damage distribution profiles to variations in boundary inflow magnitude *g.* The stationary distributions of damage are plotted for the range of boundary inflow values g∈{5,4,3,2,1.5} to evaluate how sensitive the lineage quality is to the boundary conditions imposed at *x* = 0. **(a)** For the stem population, the damage distributions exhibit a slight rightward shift and increased sharpness with reduced peak values as the inflow parameter *g* decreases. **(b)** For the TD population, the damage profiles remain nearly invariant despite the variation in *g*. The invariance shows that the TD population is effectively insensitive to fluctuations in the boundary conditions applied to the cell population.

Fig 14 shows the ratio dynamics. All nonhomogeneous inflows induce transient overshoot, with amplitudes of similar scale and a maximum at intermediate *g*. The corresponding steady-state damage profiles are shown in Fig 15. Fig 15a shows the stem distributions, which shift slightly rightward and sharpen as *g* decreases, while Fig 15b shows the TD distributions, which remain nearly invariant.

Table 8 summarizes quantitative metrics. As *g* decreases from 5 to 1.5, stem averages decline modestly from 0.1011 to 0.0946 and rightmost supports contract from 0.8850 to 0.7200. By contrast, TD averages remain nearly fixed between 0.2572 to 0.2581 and supports remain unchanged. The overshoot magnitude *R* is non-monotone in *g*, peaking at intermediate inflows.

**Table 8:** Steady metrics for *P* and *W* and overshoot *R* across boundary inflows. Stem and TD damage metrics vary only slightly with *g*, and overshoot remains modest, peaking at intermediate *g*.

g	Stem metrics			TD metrics			R
	$\langle x\rangle_{P}$	$x_{P}^{m}$	$x_{P}^{r}$	$\langle x\rangle_{W}$	$x_{W}^{m}$	$x_{W}^{r}$	
5	0.1011	0.0550	0.8850	0.2572	0.1300	1.4750	0.1666
4	0.0991	0.0600	0.8300	0.2575	0.1250	1.4750	0.1885
3	0.0973	0.0600	0.7800	0.2578	0.1250	1.4750	0.1873
2	0.0954	0.0650	0.7400	0.2580	0.1200	1.4750	0.1636
1.5	0.0946	0.0650	0.7200	0.2581	0.1200	1.4750	0.1406

Our results show that boundary inflows mainly affect transient dynamics and sharpen the stem damage profile. Long-term damage metrics remain unchanged, confirming that the model’s conclusions are robust to boundary conditions.

#### Partitioning parameter sweep: Robustness to asymmetric partitioning.

Finally, we vary the partitioning fractions that determine how damage is segregated between daughter cells at division. The baseline assumes symmetric partitioning for self-renewal with $\alpha_{1}=\alpha_{2}=0.5$ and differentiation with $\beta_{1}=\beta_{2}=0.5$, and a mildly asymmetric split for asymmetric divisions with $\gamma_{1}=1/3$ and $\gamma_{2}=2/3$. Experimental evidence, however, suggests that stem daughters often inherit disproportionately less damage than TD daughters. To systematically study different damage distribution rules, we first vary $\alpha_{1},\beta_{1}\in \{0.1,0.3,0.5\}$ with $\gamma_{1}=1/3,\gamma_{2}=2/3$, and then sweep $\gamma_{1}\in \{0.1,0.3,0.5,0.7,0.9\}$ with fixed $\alpha_{1}=\beta_{1}=0.5$.

**Fig 16:**
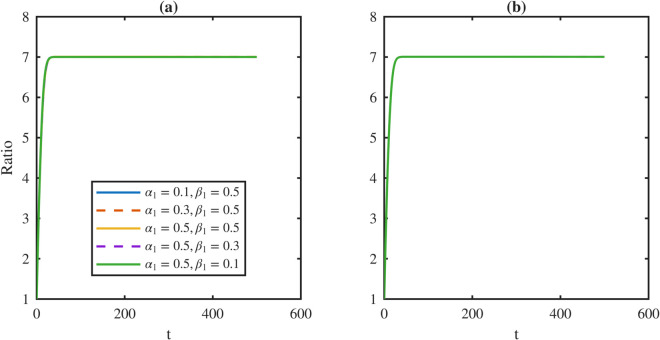
Robustness of transient population dynamics to variations in damage partitioning asymmetry. The temporal evolution of the TD-to-stem ratio $\bar{W}/\bar{P}$ is plotted to evaluate the sensitivity of lineage dynamics to the parameters governing damage segregation during cell division. **(a)** Sensitivity to primary weights: trajectories for varying partitioning fractions $\alpha_{1},\beta_{1}\in \{0.1,0.3,0.5\}$. **(b)** Sensitivity to auxiliary weights: trajectories for varying auxiliary partitioning fractions $\gamma_{1}\in \{0.1,0.3,0.5,0.7,0.9\}$. In both panels, the curves overlap almost perfectly. The overlap shows that the macroscopic population ratio dynamics are highly robust and largely insensitive to the specific mathematical tuning of the partitioning asymmetry.

Fig 16 shows the TD-to-stem ratio dynamics. Fig 16a corresponds to $\alpha_{i},\beta_{i}$ variations, while Fig 16b corresponds to $\gamma_{i}$ variations. Across both sweeps, the trajectories are virtually indistinguishable. There is no overshoot or oscillation, demonstrating complete robustness in contrast to the strong sensitivities seen under TDD or when asymmetric-division probability varies.

**Fig 17:**
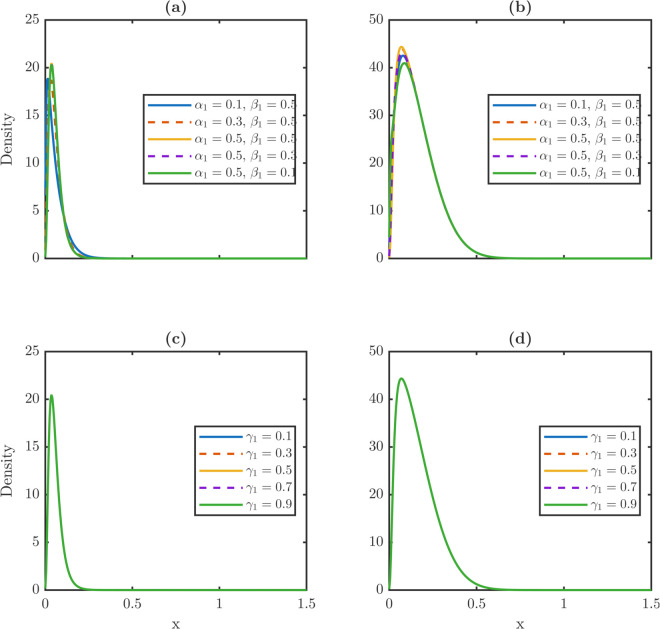
Insensitivity of steady-state damage density profiles to partitioning asymmetry parameters. Stationary damage distributions for the stem and TD populations are analyzed to determine the impact of damage segregation mechanics on lineage quality. **(a,b)** Varying primary weights: steady-state damage profiles for stem (a) and TD (b) cells under varying partitioning fractions $\alpha_{1},\beta_{1}\in \{0.1,0.3,0.5\}$. **(c,d)** Varying auxiliary weight: steady-state damage profiles for stem (c) and TD (d) cells under varying auxiliary fractions $\gamma_{1}\in \{0.1,0.3,0.5,0.7,0.9\}$. Across all parameter sweeps, the damage distribution profiles remain virtually unchanged. This indicates that while partitioning asymmetry is a key biological feature of the model, its quantitative variation exerts only minor effects on the final damage distribution. The result contrasts with the strong sensitivities induced by dedifferentiation mechanisms, as seen in earlier figures.

The stationary distributions in Fig 17 reinforce this robustness. Fig 17a and 17b show stem and TD distributions under $\alpha_{i},\beta_{i}$ variations, while Fig 17c and 17d show the corresponding distributions under $\gamma_{i}$ variations. In all cases, stem and TD profiles are nearly identical, with no visible shifts in averages, maxima, or rightmost supports.

Table 9 quantifies this robustness. In the α,β sweep, stem averages remain fixed near 0.059, TD averages near 0.163. Except for $x_{P}^{r}$, maximum and rightmost supports vary only in the second decimal place, and overshoot fractions are negligible with $R\sim 10^{-4}$. The *γ* sweep yields equally invariant metrics, with *R*<10^−3^ for all $\gamma_{1}\in [0.1,0.9]$.

**Table 9:** Steady damage metrics and overshoot *R* under variations in $\alpha_{1}$, $\beta_{1}$, and $\gamma_{1}$. For $\alpha_{1},\beta_{1}$ sweeps, $\gamma_{1}=1/3$; for $\gamma_{1}$ sweep, $\alpha_{1}=\beta_{1}=0.5$. Metrics are stable up to numerical precision; *R*<10^−3^.

$\alpha_{1}$	$\beta_{1}$	$\gamma_{1}$	Stem metrics			TD metrics			*R*
			$\langle x\rangle_{P}$	$x_{P}^{m}$	$x_{P}^{r}$	$\langle x\rangle_{W}$	$x_{W}^{m}$	$x_{W}^{r}$	
0.1	0.5	1/3	0.0587	0.0200	0.5450	0.1626	0.0850	0.9550	$5.23\times 10^{-4}$
0.3	0.5	1/3	0.0585	0.0350	0.4600	0.1630	0.0750	0.9550	$9.46\times 10^{-4}$
0.5	0.5	1/3	0.0585	0.0400	0.4500	0.1631	0.0750	0.9550	$9.22\times 10^{-4}$
0.5	0.3	1/3	0.0586	0.0400	0.4500	0.1628	0.0800	0.9550	$3.26\times 10^{-4}$
0.5	0.1	1/3	0.0590	0.0400	0.4550	0.1619	0.0900	0.9550	$4.05\times 10^{-4}$
0.5	0.5	0.1	0.0585	0.0400	0.4500	0.1631	0.0750	0.9550	$9.61\times 10^{-4}$
0.5	0.5	0.3	0.0585	0.0400	0.4500	0.1631	0.0750	0.9550	$9.57\times 10^{-4}$
0.5	0.5	0.5	0.0585	0.0400	0.4500	0.1631	0.0750	0.9550	$9.55\times 10^{-4}$
0.5	0.5	0.7	0.0585	0.0400	0.4500	0.1631	0.0750	0.9550	$9.56\times 10^{-4}$
0.5	0.5	0.9	0.0585	0.0400	0.4500	0.1631	0.0750	0.9550	$9.58\times 10^{-4}$

Partitioning asymmetry has a negligible impact on either transient or steady-state damage distributions. By contrast, TDD and division probabilities strongly reshape overshoots and oscillations. The model is therefore robust to moderate asymmetries: directing damage preferentially toward TD daughters enhances stem protection without destabilizing TD populations. Biologically, the preferential segregation of damage has been observed experimentally as a stabilizing mechanism in the long-lived stem population.

Collectively, these robustness checks show that the qualitative behaviors of the system persist across a broad parameter range, reinforcing the stability of our conclusions.

### Biological insights and mechanistic interpretation

The PDE simulations highlight how dedifferentiation modulates damage flow, linking mathematical regimes to biological observations of stem cell plasticity and cancer risk. Such connections situate our computational findings within the broader biological context.

Partitioning during stem-cell division emerges as the primary stabilizer of long-term tissue maintenance. In baseline simulations, the absence of partitioning causes unbounded damage drift. Total cell numbers remain conserved, but the stem population eventually collapses. Thus, nonlocal partitioning is indispensable for maintaining bounded homeostasis.

TDD functions as an effective damage filter. Under constant dedifferentiation, all TD cells—regardless of damage—can revert to the stem population, which accelerates stem aging. In contrast, TDD activates only when TD damage exceeds a critical threshold *x*_*c*_. Cells returning at high *x* possess poor fitness, fail to self-renew effectively. They quickly redifferentiate and are eliminated by the damage-dependent death rate δ(x). The routing of highly damaged cells through the stem state establishes a detoxifying loop. Heavily damaged cells are transiently cycled through the stem population before apoptotic clearance, whereas low-damage TD cells continue along their normal differentiation pathway. Through this filtering mechanism, TDD protects stem-cell quality even under sustained dedifferentiation pressure.

Partial repair provides an additional protective mechanism. If dedifferentiation reduces the inherited damage to a fraction ρx with 0<ρ<1, reverting cells re-enter the stem population with a lighter damage burden. Even modest levels of repair prevent excessive accumulation of damage, thereby stabilizing stem-cell homeostasis. The protective effect is biologically plausible, since reprogramming and dedifferentiation often trigger epigenetic remodeling and repair pathways. Consequently, repair-modulated dedifferentiation represents a realistic mechanism for recycling cells while avoiding the import of excessive damage into the stem population.

While formulated mathematically, the mechanisms of TDD and partial repair capture specific biological stress responses. Biologically, TDD parallels the phenomenon where mature cells—such as pancreatic *β*-cells under glucotoxic stress [57] or Schwann cells following nerve injury [58]—revert to a progenitor state. Implicitly, these reversions happen only when cellular damage signaling exceeds a critical homeostatic capacity. This threshold is further identified in the intestinal epithelium. Experimental study demonstrates that Dll 1 precursors dedifferentiate into Lgr5 stem cells following stem cell population ablation by irradiation [59]. However, they rarely generate signature ribbons that Lgr5 stem cells typically form under normal conditions. The prevention of the dedifferentiation of mouse secretory cells by only a single stem cell suggests that stem cells inhibit dedifferentiation [5]. This inhibition can be lifted only when the stem population is completely ablated [60], confirming the existence of the dedifferentiation threshold.

Similarly, the partial repair term in our model serves as a phenomenological proxy for intracellular rejuvenation mechanisms activated during state transitions. These include the asymmetric segregation of protein aggregates, aggresomes, during cell division [61], which allows cells to manage proteotoxic load. Additionally, it represents cellular reprogramming by expression of Oct4, Sox2, Klf4, and c-Myc (OSKM) to ameliorate cellular aging processes and extend organismal lifespan in mice with premature aging [62]. It has been documented that dedifferentiation into an induced pluripotent cell process is related to mitophagy-induced rejuvenation [63]. This process is achieved by mitochondrial clearance through autophagy following mitochondrial fission. However, the relationship between dedifferentiation and damage repair is more complex. Transit cellular senescence and acute inflammatory responses induce cellular dedifferentiation by repairing damaged tissues [60].

Asymmetric division functions mainly as a transient destabilizer. Varying the probability of asymmetric division *p*_3_ induces overshoot and oscillatory dynamics in the TD-to-stem ratio, with the strongest effects observed at intermediate values of *p*_3_. However, long-term damage statistics remain robust, indicating that asymmetric division perturbs transient dynamics without compromising overall homeostasis.

Boundary inflows and partitioning asymmetries are found to be negligible perturbations. Introducing inflows of minimally damaged cells sharpens stem damage distributions slightly but does not alter long-term steady states. Similarly, sweeping partition fractions across a broad range produces nearly identical damage distributions. These results confirm the system’s robustness to moderate asymmetries. The robustness is consistent with experimental findings that dividing cells asymmetrically segregate aging factors away from stem daughter cells to maintain lineage stability and integrity.

Collectively, the simulations and biological implications establish a hierarchy of stabilizing mechanisms within the model. Partitioning is indispensable for achieving bounded homeostasis. TDD acts as a damage filter that prevents stem collapse, while partial repair further alleviates the damage flux burden. By contrast, asymmetric division primarily influences transient dynamics, and partition asymmetries exert only minor long-term effects. The synthesis of these findings is consistent with experimental evidence. The consistency reinforces the view that plasticity, partitioning, damage filtering, and repair jointly govern tissue renewal and the emergence of malignancy.

## Discussion

This study examines how dedifferentiation shapes long-term tissue dynamics within a damage-structured PDE framework. Our results highlight a context-dependent duality. Dedifferentiation is not inherently beneficial or detrimental. But its outcome—whether it rescues homeostasis or drives aging—is determined by the presence of filtering mechanisms like TDD or damage repair. We showed that partitioning, threshold filtering, and partial repair govern whether plasticity acts as a regenerative safeguard or accelerates decline. This duality reconciles divergent findings. Melanocyte stem cells benefit from dedifferentiation for maintenance [6]. However, dedifferentiation-derived neural stem cells may import damage and predispose to malignancy [9]. Our model extends prior observations of stem-cell plasticity by providing quantitative conditions under which dedifferentiation is stabilizing versus pathological.

The implications reach regenerative medicine and oncology. Dedifferentiation thresholds and repair efficiency emerge as control points. Higher thresholds reduce malignant re-entry of damaged cells, while enhanced repair preserves regenerative capacity. Thus, the contribution of dedifferentiation is defined not by the process itself, but by the rigor of the filtering mechanisms that regulate it.

Methodologically, we introduced nonlocal *δ*-kernel partitioning, a conservative finite-volume discretization, and damage-distribution metrics. These tools ensure conservation and reproducibility while linking microscopic rules of partitioning and repair to tissue-level outcomes.

Beyond structural simplifications discussed in the Methods section, our numerical scheme is first-order accurate—sufficient here but limited for long-time simulations or sharper transients. Higher-order schemes, such as WENO [64], and Runge–Kutta or adaptive stepping could improve accuracy. Mortality was modeled linearly, underestimating nonlinear apoptosis and malignant initiation at high lesion loads. The deterministic mean-field framework omits stochasticity in division, damage inheritance, and dedifferentiation, which real tissues use to shape resilience. Extensions to stochastic PDE or agent-based models would capture such variability. Likewise, our reduced two-compartment lineage excluded TA cells and quiescence, though the framework can be extended.

Further insights could come from global sensitivity analysis by Latin hypercube sampling or the Morris method. These methods allow us to assess parameter interactions and their influence on key outcomes such as steady-state damage or population ratios. Notably, our results indicate that the dedifferentiation rate $\lambda_{R}$, threshold *x*_*c*_, and retention fraction *ρ* are particularly sensitive and merit experimental validation.

Despite these limitations, simulations were robust under grid refinement and parameter sweeps, with exact mass conservation. Key findings—such as partitioning being essential for bounded damage and dedifferentiation acting as a double-edged process—are likely to persist in more complex models.

In sum, dedifferentiation functions as a regenerative safeguard when coupled with filtering or repair, but becomes a liability when applied indiscriminately. Our work quantifies this trade-off within a reproducible PDE framework. As a result, our work integrates mathematical modeling with experimental biology. At the same time, it provides a foundation for strategies that harness stem-cell plasticity to support regeneration while minimizing risks of aging and cancer.

## Appendix

To support the main analysis of the duality of dedifferentiation in the PDE model, we detail auxiliary derivations. These derivations include scaling laws for steady states and extensions incorporating stem attrition and quiescence.

## Two-step control strategy

In this section, we present a two-step control strategy for the system. Specifically, the parameters $p_{1},p_{2},$ and $\lambda_{P}$ are adjusted while all other parameters are held fixed to achieve prescribed steady states. This control strategy guides the choice of $p_{1},p_{2},$ and $\lambda_{P}$ in numerical simulations so that the TD-to-stem ratio matches the target value with $\bar{W}=10$. To formalize this approach, we consider the reduced population dynamics in Eq (6), together with the complete control strategies from Eqs (8)–(11) and a constant death rate δ(x)≡δ>0:

$$\left\{ \begin{array}{cc} \frac{d\bar{P}}{dt} & =(2f(t)-1)\lambda_{P}(t)\bar{P}+\lambda_{R}(t)\bar{W},\\ \frac{d\bar{W}}{dt} & =(2-2f(t))\lambda_{P}(t)\bar{P}-\lambda_{R}(t)\bar{W}(t)-\delta \bar{W}, \end{array}\right.$$ (14)

At steady state $\left({\bar{P}}^{*},{\bar{W}}^{*}\right)$ with distributions P^*^(*x*),W^*^(*x*), the system reduces to


$$\left\{ \begin{array}{cc} 0 & =(2f({\bar{W}}^{*})-1)\lambda_{P}({\bar{W}}^{*}){\bar{P}}^{*}+\lambda_{R}{\bar{W}}^{*},\\ 0 & =(2-2f({\bar{W}}^{*}))\lambda_{P}({\bar{W}}^{*}){\bar{P}}^{*}-\lambda_{R}{\bar{W}}^{*}-\delta {\bar{W}}^{*}. \end{array}\right.$$

Summing the equations gives

$$\lambda_{P}({\bar{W}}^{*}){\bar{P}}^{*}=\delta {\bar{W}}^{*},\;⟹\;\frac{{\bar{P}}^{*}}{{\bar{W}}^{*}}=\frac{\delta }{\lambda_{P}({\bar{W}}^{*})}.$$ (15)

Thus, the steady-state ratio ${\bar{P}}^{*}/{\bar{W}}^{*}$ is fixed by the balance of replication and death, while the absolute sizes depend on feedback strengths.

**Proposition 1** (Scaling law of the steady state). A*ssume Hill-type feedback*


$$p_{j}(\bar{W})=\frac{{\hat{p}}_{j}}{1+(k_{j}\bar{W}{)}^{m_{j}}},\;\lambda_{P}(\bar{W})=\frac{{\hat{\lambda }}_{P}}{1+(k_{3}\bar{W}{)}^{m_{3}}},\;\lambda_{R}(\bar{P})=\frac{{\hat{\lambda }}_{R}}{1+(k_{4}\bar{P}{)}^{m_{4}}}.$$

*Fix all parameters except $k_{1},k_{2},k_{3},k_{4}$. If the steady state $\left({\bar{P}}^{*},{\bar{W}}^{*}\right)$ of the unscaled system is unique, then for any A>0 the system with scaled feedback constants $k_{i}^{\prime }=Ak_{i}$ admits the rescaled steady state*


$${𝒮}^{\prime }=\left(\frac{{\bar{P}}^{*}}{A},\;\frac{{\bar{W}}^{*}}{A}\right).$$

We state the result under the explicit assumption of the uniqueness of the steady state. If multiple equilibria exist the same scaling $k_{i}\mapsto Ak_{i}$ induces a one-to-one correspondence between equilibria of the original and rescaled systems via $\left(\bar{P},\bar{W})\mapsto (\bar{P}/A,\bar{W}/A\right)$.

*Proof:* Let $\left({\bar{P}}^{*},{\bar{W}}^{*}\right)$ satisfy the steady-state total-mass balance

$$0=(p_{1}({\bar{W}}^{*})-p_{2}({\bar{W}}^{*}))\;\lambda_{P}({\bar{W}}^{*})\;{\bar{P}}^{*}+\lambda_{R}({\bar{P}}^{*})\;{\bar{W}}^{*},$$ (16)

$$0=(1-p_{1}({\bar{W}}^{*})+p_{2}({\bar{W}}^{*}))\;\lambda_{P}({\bar{W}}^{*})\;{\bar{P}}^{*}-(\delta +\lambda_{R}({\bar{P}}^{*}))\;{\bar{W}}^{*}.$$ (17)

For fixed *A*>0 define scaled Hill parameters $k_{i}^{\prime }=Ak_{i}$ and let $p_{j}^{\prime },\lambda_{P}^{\prime },\lambda_{R}^{\prime }$ denote the corresponding Hill functions. By direct algebra of the Hill expressions, one has the identities


$$p_{j}^{\prime }\;\left(\frac{\bar{W}}{A}\right)=p_{j}(\bar{W}),\;\lambda_{P}^{\prime }\;\left(\frac{\bar{W}}{A}\right)=\lambda_{P}(\bar{W}),\;\lambda_{R}^{\prime }\;\left(\frac{\bar{P}}{A}\right)=\lambda_{R}(\bar{P}),$$

for all $\bar{P},\bar{W}$ and j=1,2. Set ${\bar{P}}^{\prime }={\bar{P}}^{*}/A$ and ${\bar{W}}^{\prime }={\bar{W}}^{*}/A$. Substituting into the left-hand side of the rescaled steady equations gives, for the first equation,


$$(p_{1}^{\prime }({\bar{W}}^{\prime })-p_{2}^{\prime }({\bar{W}}^{\prime }))\lambda_{P}^{\prime }({\bar{W}}^{\prime }){\bar{P}}^{\prime }+\lambda_{R}^{\prime }({\bar{P}}^{\prime }){\bar{W}}^{\prime }=\frac{1}{A}((p_{1}({\bar{W}}^{*})-p_{2}({\bar{W}}^{*}))\lambda_{P}({\bar{W}}^{*}){\bar{P}}^{*}+\lambda_{R}({\bar{P}}^{*}){\bar{W}}^{*}),$$

which vanishes by (16). The second equation yields an identical factor 1/*A* times the left-hand side of (17) and hence also vanishes. Thus $\left({\bar{P}}^{\prime },{\bar{W}}^{\prime }\right)$ is an equilibrium of the rescaled system. Uniqueness of the steady state in the rescaled system implies this equilibrium is the unique rescaled steady state, proving the claimed scaling. □

**Remark 2.** *The scaling law provides a two-step control strategy: fix the population ratio through replication–death balance, then adjust absolute population sizes by uniform rescaling of k*_*i*_.

## Numerical scheme

For completeness, we provide the detailed formulation of the discretization scheme.

### Discretization strategy

We discretize the PDE system by an upwind finite-volume scheme in space and forward Euler in time. Nonlocal partitioning terms are computed by conservative linear interpolation, which attains second-order accuracy in space. Because the transport term is approximated with a first-order upwind scheme, and time integration relies on forward Euler, the overall method remains first-order accurate. Stability requires the standard CFL condition


$$\Delta t\leq C\;\frac{\Delta x}{\max\{v_{P},v_{W}\}},\;0<C<1.$$

The scheme is first-order in time, with local truncation error 𝒪(Δx+Δt), and preserves non-negativity.

### Fully discrete scheme

On a finite domain x∈[0,A], let $x_{i}=i\Delta x$ (i=0,…,N) and $t_{n}=n\Delta t$. We denote discrete states by $P_{i}^{n}\approx P(t_{n},x_{i})$ and $W_{i}^{n}\approx W(t_{n},x_{i})$. The update equations are

$$\left\{ \begin{array}{cc} P_{i}^{n+1} & =P_{i}^{n}-\frac{v_{P}\Delta t}{\Delta x}(P_{i}^{n}-P_{i-1}^{n})+\Delta t[\frac{p_{1}\lambda_{P}}{\alpha_{1}}P(t_{n},x_{i}/\alpha_{1})+\frac{p_{1}\lambda_{P}}{\alpha_{2}}P(t_{n},x_{i}/\alpha_{2})\\ & \;+\frac{p_{3}\lambda_{P}}{\gamma_{1}}P(t_{n},x_{i}/\gamma_{1})-\lambda_{P}P_{i}^{n}+\lambda_{R}W_{i}^{n}],\\ W_{i}^{n+1} & =W_{i}^{n}-\frac{v_{W}\Delta t}{\Delta x}(W_{i}^{n}-W_{i-1}^{n})+\Delta t[\frac{p_{2}\lambda_{P}}{\beta_{1}}P(t_{n},x_{i}/\beta_{1})+\frac{p_{2}\lambda_{P}}{\beta_{2}}P(t_{n},x_{i}/\beta_{2})\\ & \;+\frac{p_{3}\lambda_{P}}{\gamma_{2}}P(t_{n},x_{i}/\gamma_{2})-(\delta +\lambda_{R})W_{i}^{n}]. \end{array}\right.$$ (18)

The CFL condition guarantees the positivity of coefficients of $P_{i}^{n}$ and $W_{i}^{n}$ in the upwind scheme in Eq (18), avoiding spurious oscillations [65].

### Interpolation for nonlocal terms

If $\alpha x_{i}=x_{j}+\omega \Delta x$ with 0≤ω<1, linear interpolation has partition-of-unity property and

$$P(t_{n},\alpha x_{i})=(1-\omega )P(t_{n},x_{j})+\omega P(t_{n},x_{j+1})$$ (19)

holds. This interpolation introduces an error of order $𝒪(\Delta x^{2})$ while maintaining the conservative property

$$\sum_{i}P(t_{n},\alpha x_{i})\Delta x=\sum_{j}P(t_{n},x_{j})\Delta x.$$ (20)

An analogous identity for *W* holds. Thus, the total mass is preserved to machine precision.

### Boundary conditions on the truncated numerical domains

In the continuum formulation, we impose homogeneous Dirichlet conditions at the low-damage boundary *x* = 0, and assume decay as x→∞. Numerically, we truncate the domain to x∈[0,A], with *A* chosen large enough that the solution has negligible mass near *x* = *A*. At the computational boundary *x* = *A*, we impose an outflow condition with zero-inflow. Since $v_{P},v_{W}>0$, all characteristics exit the domain, and no boundary conditions are required. Here, characteristics denote the curves along which the PDE solution is transported, representing single-cell damage trajectories that connect individual fates with population-level distributions.

In the finite-volume scheme, the conservative update for cell averages is


$$\left\{ \begin{array}{cc} P_{i}^{n+1} & =P_{i}^{n}-\frac{\Delta t}{\Delta x}(F_{i+1/2}^{n}-F_{i-1/2}^{n})+\Delta t\;S_{i}^{n},\\ W_{i}^{n+1} & =W_{i}^{n}-\frac{\Delta t}{\Delta x}(G_{i+1/2}^{n}-G_{i-1/2}^{n})+\Delta t\;T_{i}^{n}. \end{array}\right.$$

Numerical fluxes are


$$F_{i+1/2}^{n}=\left\{ \begin{array}{cc} v_{P}P_{i}^{n}, & v_{P}\geq 0,\\ v_{P}P_{i+1}^{n}, & v_{P}<0, \end{array}\right.\;G_{i+1/2}^{n}=\left\{ \begin{array}{cc} v_{W}W_{i}^{n}, & v_{W}\geq 0,\\ v_{W}W_{i+1}^{n}, & v_{W}<0, \end{array}\right.$$

and source terms $S_{i}^{n},T_{i}^{n}$ given by the nonlocal division rules. At the right boundary interface *x*_*N*_ = *A*, the fluxes reduce to


$$F_{N+1/2}^{n}=v_{P}P_{N}^{n},\;G_{N+1/2}^{n}=v_{W}W_{N}^{n},$$

which depend only on the last interior states. This formulation enforces a discrete outflow boundary condition: all mass leaving the domain exits through *x* = *A*, with no artificial inflow. The resulting scheme preserves global mass balance up to source and sink terms.

### Pseudocode summary

For clarity and reproducibility, the algorithmic structure of the scheme is summarized below. This pseudocode mirrors our MATLAB implementation with Matlab version R2025a Update 1. It also illustrates one full timestep update for *P* and *W*, including flux evaluation, interpolation of nonlocal terms, and reaction updates:

**Algorithm 1. Finite-volume update for stem (*P*) and TD (*W*) populations.**



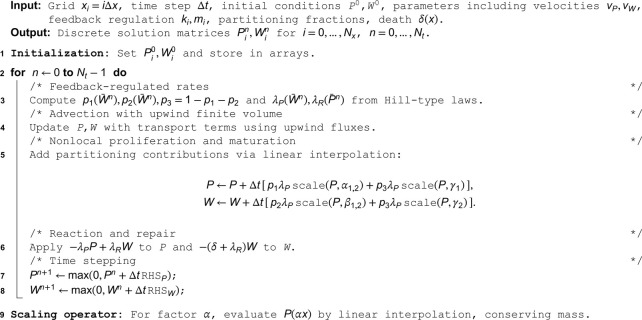



## Intermediate transit-amplifying cells and hierarchical complexity

Our current model utilizes a minimal two-compartment framework, distinguishing only between long-lived stem cells and functional TD cells. We acknowledge that this is a simplification of the biological reality, where an intermediate population of TA cells often serves as a bridge. TA cells typically exhibit rapid cycling and limited self-renewal capacity, functioning to amplify the cellular output generated by scarce stem cells before terminal differentiation [45]. A three-compartment hierarchy from stem to TA to TD would offer a more plausible representation. However, we argue that the qualitative insights of our two-compartment model remain robust, particularly regarding the trade-offs of dedifferentiation. The inclusion of a TA compartment would likely impact the system dynamics in three key ways:

1. **Timescale Separation and Effective Yield:** TA cells operate on a much faster timescale than stem cells. In many mathematical contexts, rapidly turning-over intermediate compartments can be “lumped” into an effective amplification factor. For instance, one stem cell division does not produce one TD cell, but rather 2^*k*^ TD cells, where *k* is the number of TA division rounds. Our model effectively captures this through the proliferation rate $\lambda_{P}$, which can be interpreted as the distinct rate of clone initiation. At the same time, the population size *W* represents the aggregate functional mass.
2. **Feedback Delays and Stability:** The TA population introduces a time delay between a stem cell decision and the resulting change in the TD population. In feedback-regulated systems, such delays are known to destabilize homeostatic states. They potentially induce oscillatory behavior via Hopf bifurcations rather than the monotonic convergence often seen in two-compartment ODEs. Thus, the introduction of the TA population would narrow the parameter space for stable non-oscillatory homeostasis. However, the fundamental capacity of dedifferentiation to rescue the lineage from extinction—our primary focus—would persist.
3. **Damage Amplification vs. Dilution:** TA cells divide rapidly, which could act as a “damage amplifier” if replication errors dominate. They can also act as a “damage diluter” if asymmetric segregation is enforced during TA divisions. However, since TA cells are transient and eventually flush out of the tissue, they do not serve as a long-term reservoir for damage accumulation in the same way stem cells do. Therefore, the long-term aging profile of the tissue is still governed by the damage burden retained in the stem population and the influx of damage via dedifferentiation. Both factors are explicitly captured in our current framework.

In summary, adding a TA population would introduce richer transient dynamics and potential instabilities due to signaling delays. However, the current two-compartment approximation captures the essential “source-sink” relationship. The relationship is required to evaluate the long-term structural stability of the lineage under damage stress. By omitting the intermediate stage, we focus on the worst-case scenario. In such a scenario, dedifferentiating cells return directly to the long-lived stem population. Therefore, our work provides a conservative estimate of the aging risks associated with plasticity.

## Stem-cell attrition

To reflect apoptosis and long-term exit from the cycling pool, we include an effective stem-cell loss rate $\delta_{P}(x)\geq 0$. We extend the two-compartment model in Eq (2) by introducing stem cell loss through an explicit death term $\delta_{P}(x)P$. The resulting PDE system is:

$$\left\{ \begin{array}{cc} \partial_{t}P+v_{P}\partial_{x}P= & -\lambda_{P}P-\delta_{P}(x)P+\lambda_{R}W\\ & +\frac{p_{1}\lambda_{P}}{\alpha_{1}}P(t,x/\alpha_{1})+\frac{p_{1}\lambda_{P}}{\alpha_{2}}P(t,x/\alpha_{2})+\frac{p_{3}\lambda_{P}}{\gamma_{1}}P(t,x/\gamma_{1}),\\ \partial_{t}W+v_{W}\partial_{x}W= & -(\delta_{W}(x)+\lambda_{R})W\\ & +\frac{p_{2}\lambda_{P}}{\beta_{1}}P(t,x/\beta_{1})+\frac{p_{2}\lambda_{P}}{\beta_{2}}P(t,x/\beta_{2})+\frac{p_{3}\lambda_{P}}{\gamma_{2}}P(t,x/\gamma_{2}). \end{array}\right.$$ (21)

Integrating Eq (21) over the damage domain x∈[0,∞) yields the following total dynamics:


$$\left\{ \begin{array}{c} \frac{d\bar{P}}{dt}=(2p_{1}+p_{3}-1)\lambda_{P}\bar{P}-\int_{0}^{\infty }\delta_{P}(x)P(t,x)\;dx+\lambda_{R}\bar{W},\\ \frac{d\bar{W}}{dt}=(2p_{2}+p_{3})\lambda_{P}\bar{P}-\int_{0}^{\infty }\delta_{W}(x)W(t,x)\;dx-\lambda_{R}\bar{W}. \end{array}\right.$$

Assuming constant death rates $\delta_{P},\delta_{W}$, and introducing the compact renewal fraction notation $f=(2p_{1}+p_{3})/2$, we obtain the reduced system:

$$\left\{ \begin{array}{c} \frac{d\bar{P}}{dt}=a\bar{P}+b\bar{W},\\ \frac{d\bar{W}}{dt}=c\bar{P}+d\bar{W}, \end{array}\right.$$ (22)

where we define the parameters:


$$a:=(2f-1)\lambda_{P}-\delta_{P},\;b:=\lambda_{R},\;c:=2(1-f)\lambda_{P},\;d:=-(\lambda_{R}+\delta_{W}).$$

This linear system summarizes the net effects, *f*, of self-renewal versus differentiation, dedifferentiation $\lambda_{R}$, and constant death rates $\delta_{P},\delta_{W}$. Setting time derivatives to zero gives:


$$a{\bar{P}}^{*}+b{\bar{W}}^{*}=0,\;c{\bar{P}}^{*}+d{\bar{W}}^{*}=0.$$

The trivial equilibrium $\left({\bar{P}}^{*},{\bar{W}}^{*})=(0,0\right)$ always exists. A nontrivial equilibrium for this homogeneous linear system exists only when the coefficient matrix is singular, i.e., when the determinant Δ:=ad−bc vanishes. If Δ=0, the two linear equations are linearly dependent and there is a one-parameter family of equilibria. Biologically, a continuum of equilibria is usually unrealistic. In practice, a unique positive steady state is generated by nonlinearities. For example, feedback of *p*_*i*_ or $\lambda_{R}$ ensures the uniqueness. This is why the full model with feedback functions $p_{i}(\bar{W})$, $\lambda_{R}(\bar{P})$ admits isolated, robust steady states. The characteristic polynomial for the Jacobian $𝒥=(\begin{array}{cc} a & b\\ c & d \end{array})$ is


$$\chi (\lambda )=\lambda^{2}-tr(𝒥)\lambda +det(𝒥),$$

and the trivial equilibrium (0,0) is locally asymptotically stable if and only if the Routh-Hurwitz conditions hold:

$$tr(𝒥)=(2f-1)\lambda_{P}-\delta_{P}-(\lambda_{R}+\delta_{W})<0,$$ (T)

$$det(𝒥)=-\left((2f-1)\lambda_{P}-\delta_{P}\right)(\lambda_{R}+\delta_{W})-2(1-f)\lambda_{P}\lambda_{R}>0.$$ (D)

A change of stability involving a zero eigenvalue occurs when Δ=0. Solving Eq (D) for the critical renewal fraction $f_{\text{crit}}$ yields:


$$f_{\text{crit}}:=\frac{\delta_{P}\delta_{W}+\delta_{P}\lambda_{R}+\delta_{W}\lambda_{P}-\lambda_{P}\lambda_{R}}{2\delta_{W}\lambda_{P}}\;=\underset{\text{original }\tilde{f}}{\underbrace{\frac{\delta_{W}-\lambda_{R}}{2\delta_{W}}}}+\frac{\delta_{P}(\delta_{W}+\lambda_{R})}{2\delta_{W}\lambda_{P}}.$$

We see that the numerator combines death-death, death-dedifferentiation, and death-replication cross terms. We also see that the sign of $\lambda_{P}\lambda_{R}$ subtracts from the numerator, which means that strong replication-dedifferentiation tends to reduce $f_{\text{crit}}$. In addition, without stem cell death, the original critical threshold appears as:


$$\tilde{f}=\frac{\delta_{W}-\lambda_{R}}{2\delta_{W}}.$$

Introducing stem cell death elevates the critical renewal parameter by $\frac{\delta_{W}+\lambda_{R}}{2\delta_{W}\lambda_{P}}\delta_{P}$, which is proportional to the stem death rate $\delta_{P}$. This additional term accounts for the need to balance the outflux induced by stem death with increased stem replication. At any nontrivial steady state (P^*^,W^*^) of Eq (21), the corresponding total $\left({\bar{P}}^{*},{\bar{W}}^{*}\right)$ satisfies $\frac{d\bar{P}}{dt}=\frac{d\bar{W}}{dt}=0$ in Eq (22). Summing the balance equations gives the steady-state ratio of stem to TD populations:


$$\frac{{\bar{P}}^{*}}{{\bar{W}}^{*}}=\frac{\delta_{W}}{\lambda_{P}-\delta_{P}}.$$

This simple expression reveals a counterintuitive prediction: increasing stem cell death $\delta_{P}$ can elevate the relative frequency of stem cells. Mechanistically, this effect arises because stem cells regulate their output through a dynamic balance of symmetric and asymmetric divisions, which is responsive to tissue context. Symmetric self-renewal tends to dominate in conditions of scarcity. Examples of scarcity include early developmental stages or recovery following injury. This dominance favors the expansion of the stem cell population. By contrast, asymmetric division is more prevalent during homeostasis in mature tissues. This division mode maintains a stable stem cell population and replenishes differentiated cells [48]. The introduction of explicit stem-cell loss effectively mimics a state of deficit. This state shifts the balance of division modes toward symmetric self-renewal and thereby drives excessive stem cell expansion despite the additional attrition.

## Stem-cell quiescence

We extend the two-compartment stem-TD system by introducing a quiescent stem cell population *Q*(*t*,*x*), representing stem cells that have temporarily exited the active cell cycle. For convenience, we denote the right-hand sides of the standard dynamics without death terms δ(x)W in Eq (2) for stem and TD cells by *S*_*P*_(*t*,*x*) and *S*_*W*_(*t*,*x*), respectively. The resulting three-compartment minimal model is given by


$$\left\{ \begin{array}{c} \partial_{t}P+v_{P}\partial_{x}P=S_{P}(t,x)-s_{P}(x)P+r_{Q}(x)Q-\delta_{P}(x)P,\\ \partial_{t}Q+v_{Q}\partial_{x}Q=s_{P}(x)P-r_{Q}(x)Q-\delta_{Q}(x)Q,\\ \partial_{t}W+v_{W}\partial_{x}W=S_{W}(t,x)-\delta_{W}(x)W. \end{array}\right.$$

Here, $\delta_{P},\delta_{Q},\delta_{W}$ denote the death rates of active stem, quiescent stem, and TD cells, respectively. The functions *s*_*P*_(*x*) and *r*_*Q*_(*x*) represent bidirectional transition rates between active and quiescent states. Integrating over the domain x∈[0,∞) yields the ODE system for the total population sizes $\bar{P},\bar{Q},\bar{W}$:

$$\left\{ \begin{array}{c} \frac{d\bar{P}}{dt}=\left[(2f-1)\lambda_{P}-\delta_{P}-s_{P}\right]\bar{P}+r_{Q}\bar{Q}+\lambda_{R}\bar{W},\\ \frac{d\bar{Q}}{dt}=s_{P}\bar{P}-(r_{Q}+\delta_{Q})\bar{Q},\\ \frac{d\bar{W}}{dt}=2(1-f)\lambda_{P}\bar{P}-(\lambda_{R}+\delta_{W})\bar{W}. \end{array}\right.$$ (23)

We denote the equilibrium of (23) by $\left({\bar{P}}^{*},{\bar{Q}}^{*},{\bar{W}}^{*}\right)$. The steady-state quiescent population is directly proportional to the active stem population, ${\bar{Q}}^{*}=\frac{s_{P}}{r_{Q}+\delta_{Q}}{\bar{P}}^{*}$.

The steady state TD population is directly related to the active stem population *P* as before:

$$2(1-f)\lambda_{P}{\bar{P}}^{*}-(\lambda_{R}+\delta_{W}){\bar{W}}^{*}=0.$$ (24)

The equilibrium relation in Eq (24) indicates that the fast-cycling TA cells induce transient population behaviors but do not influence long-term population dynamics. Therefore, we seek the quasi-steady-state approximation where we replace $\bar{Q}$ in the $\bar{P}$ equation in Eq (23) by the steady state quiescent cell number ${\bar{Q}}^{*}=\frac{s_{P}}{r_{Q}+\delta_{Q}}{\bar{P}}^{*}$. We obtain the following reduced two-compartment stem and TD system:


$$\left\{ \begin{array}{c} \frac{d\bar{P}}{dt}=\left[(2f-1)\lambda_{P}-\delta_{P}-\frac{s_{P}\delta_{Q}}{r_{Q}+\delta_{Q}}\right]\bar{P}+\lambda_{R}\bar{W},\\ \frac{d\bar{W}}{dt}=2(1-f)\lambda_{P}\bar{P}-(\lambda_{R}+\delta_{W})\bar{W}. \end{array}\right.$$

Repeating the stability analysis for the stem cell attrition case, a change of stability happens when $f=f_{crit,q}=f_{crit}\;+\;\Delta f$. Here,


$$f_{\text{crit}}:=\frac{\delta_{P}\delta_{W}+\delta_{P}\lambda_{R}+\delta_{W}\lambda_{P}-\lambda_{P}\lambda_{R}}{2\delta_{W}\lambda_{P}}$$

is such that the change of stability occurs when $f=f_{crit}$ in the stem cell attrition case, and


$$\Delta f=\frac{s_{P}\delta_{Q}}{r_{Q}+\delta_{Q}}\frac{\lambda_{R}+\delta_{W}}{2\lambda_{P}\delta_{W}}.$$

This formula quantifies the compensatory increase in stem cell self-renewal needed to offset the loss of stem cells that enter the quiescent state and die rather than reactivating.

We analyze each parameter *s*_*P*_, $\delta_{Q}$, and *r*_*Q*_, and give its biological implications. (i) The additional Δf is proportional to *s*_*P*_. *s*_*P*_ represents the rate at which active stem cells *P* exit the cell cycle and enter the quiescent population *Q*. Since the quiescent cells are susceptible to death before they can reactivate, entering quiescence creates a leak in the stem cell population. Therefore, a higher entry rate into quiescence increases the extinction risk of the lineage. The increased risk necessitates a proportional increase in the self-renewal probability Δf of the remaining active stem cells to compensate for this loss. (ii) Δf is increasing in $\delta_{Q}$ via $\frac{\delta_{Q}}{r_{Q}+\delta_{Q}}$. This term represents the probability that a quiescent cell dies rather than returning to the active stem population. If quiescent cells are fragile or prone to apoptosis, the burden on the active stem population increases. If $\delta_{Q}=0$, then Δf=0, meaning that quiescence would have no impact on the long-term stability condition. (iii) Δf is decreasing in *r*_*Q*_ via the denominator $r_{Q}+\delta_{Q}$. *r*_*Q*_ is the rate at which quiescent cells wake up and return to the active stem population. A faster reactivation rate reduces the time cells spend in the quiescent state, thereby increasing the likelihood they survive to divide again.

Therefore, quiescence acts as a net sink on the population if there is any death in the quiescent state $\delta_{Q}>0$. To maintain homeostasis and offset the quiescence burden, the active stem cells must shift their behavior toward higher self-renewal Δf>0.
